# Signatures of criticality arise from random subsampling in simple population models

**DOI:** 10.1371/journal.pcbi.1005718

**Published:** 2017-10-03

**Authors:** Marcel Nonnenmacher, Christian Behrens, Philipp Berens, Matthias Bethge, Jakob H. Macke

**Affiliations:** 1 Research Center caesar, an associate of the Max Planck Society, Bonn, Germany; 2 Max Planck Institute for Biological Cybernetics, Tübingen, Germany; 3 Bernstein Center for Computational Neuroscience, Tübingen, Germany; 4 Centre for Integrative Neuroscience, University of Tübingen, Tübingen, Germany; 5 Institute for Ophthalmic Research, University of Tübingen, Tübingen, Germany; 6 Institute of Theoretical Physics, University of Tübingen, Tübingen, Germany; UCL, UNITED KINGDOM

## Abstract

The rise of large-scale recordings of neuronal activity has fueled the hope to gain new insights into the collective activity of neural ensembles. How can one link the statistics of neural population activity to underlying principles and theories? One attempt to interpret such data builds upon analogies to the behaviour of collective systems in statistical physics. Divergence of the specific heat—a measure of population statistics derived from thermodynamics—has been used to suggest that neural populations are optimized to operate at a “critical point”. However, these findings have been challenged by theoretical studies which have shown that common inputs can lead to diverging specific heat. Here, we connect “signatures of criticality”, and in particular the divergence of specific heat, back to statistics of neural population activity commonly studied in neural coding: firing rates and pairwise correlations. We show that the specific heat diverges whenever the average correlation strength does not depend on population size. This is necessarily true when data with correlations is randomly subsampled during the analysis process, irrespective of the detailed structure or origin of correlations. We also show how the characteristic shape of specific heat capacity curves depends on firing rates and correlations, using both analytically tractable models and numerical simulations of a canonical feed-forward population model. To analyze these simulations, we develop efficient methods for characterizing large-scale neural population activity with maximum entropy models. We find that, consistent with experimental findings, increases in firing rates and correlation directly lead to more pronounced signatures. Thus, previous reports of thermodynamical criticality in neural populations based on the analysis of specific heat can be explained by average firing rates and correlations, and are not indicative of an optimized coding strategy. We conclude that a reliable interpretation of statistical tests for theories of neural coding is possible only in reference to relevant ground-truth models.

## Author summary

Understanding how populations of neurons collectively encode sensory information is one of the central goals of computational neuroscience. In physics, systems are often characterized by identifying and describing critical points (e.g. the transition between two states of matter). The success of this approach has inspired a series of studies to search for analogous phenomena in nervous systems, and has lead to the hypothesis that these might be optimized to be poised at ‘thermodynamic critical points’. However, translating concepts from thermodynamics to neural data analysis has been a challenging endeavour. We here study the data analysis approaches that have been used to provide evidence for criticality in the brain. We find that observing signatures of criticality is closely linked to observing activity correlations between neurons– a ubiquitous phenomenon in neural data. Our study questions the experimental evidence that neural systems are optimised to exhibit thermodynamic critical behaviour. Finally, we provide practical, open-source tools for analyzing large-scale measurements of neural population activity using maximum entropy models.

## Introduction

Recent advances in neural recording technology [1, 2] and computational tools for describing neural population activity [3] make it possible to empirically examine the statistics of large neural populations and search for principles underlying their collective dynamics [4]. One hypothesis that has emerged from this approach is the idea that neural populations might be poised at a thermodynamic critical point [5, 6, 7], and that this might have consequences for how neural populations process sensory information [7, 8]. As similar observations have been made in other biological systems [9, 10, 11], it has been suggested that this might reflect a more general organising principle [12]. Critical phenomena play a central role in physics: Phase transitions mark a special point in which media qualitatively change their properties by transitioning from one state of matter into another (e.g. liquid to gaseous at boiling point, ferro-magnetic and paramagnetic phases, or the emergence of super-conductivity). As such, the behaviour of a system at critical points is informative about its intrinsic properties. Moreover, critical points are ‘special’ in the sense that they classically only occupy a small portion of the parameter space. Thus, observing that a system is constantly poised at a critical point would be surprising, and would hint at an underlying organizing mechanism that keeps the system at this point. Given the fundamental importance of critical phenomena in physics, and their success in revealing the laws the determine the behaviour of physical systems, the hypothesis that these approaches might also shed lights on principles underlying neural coding is intriguing.

Evidence in favour of this hypothesis has been put forward by a series of studies which measured neural activity from large populations of retinal ganglion cells and reported that their statistics resemble those of physical systems at a critical point [7, 8]. To this end, Tkačik and colleagues developed a data analysis framework to search for signatures of criticality in experimentally obtained measurements. Using large-scale multielectrode array recordings [2] and maximum entropy models [13, 14, 15, 16, 17, 12, 3, 18], it was observed that the normalized variance of log-probabilities diverges as a function of population size. Importantly, this quantity is mathematically equivalent to the specific heat capacity, an important characteristic which diverges at critical points. In addition, when an artificial ‘temperature’ parameter was introduced, specific heat appeared to be maximal for the statistics of the observed data, rather than for statistics which have been perturbed by changing the temperature parameter. These properties of retinal populations resemble the behaviour of physical systems at critical points. It has been hypothesised [12, 7] that the system needs to be optimized to keep itself at a critical point, for example through adaptation to stimulus statistics [19, 20, 21] or alternative mechanisms of self-organization [22, 23, 24].

A competing hypothesis states that instead generic mechanisms are sufficient to give rise to activity data with divergent specific heat, and that the presence of signatures of criticality does not provide evidence for retinal circuits being poised at a special state that is advantageous for coding. A series of theoretical studies [25, 26, 27, 28] has shown that common input (i.e,. the presence of latent variables) can account for signatures of criticality: In particular, Schwab et al [26] and Aitchison et al [27, 28] showed that Zipf scaling (an alternative characterization of criticality) and the divergence of the specific heat are closely related, and that in high-dimensional models with a low-dimensional latent variable, the specific heat diverges with system size under a wide range of circumstances [27, 28]. Similarly, it has been shown empirically that a purely feedforward model can capture Zipf-like scaling in recordings from the salamander retina [29].

Interpreting findings of thermodynamic criticality for neural populations, identifying their mechanistic underpinnings, and clarifying their relationship with alternative theories, has been fraught with difficulty. We hypothesize that this difficulty stems from a subtle but crucial difference between how the scaling behaviour of system properties is studied in thermodynamics and in practical neural data analysis: Most theoretical approaches study how system properties scale as the size of the system, *n*, is varied. In contrast, in practical neural data analysis, different “*n*” do not correspond to different system sizes, but are obtained by subsampling neural populations from a large recording (which is itself a subsample of the underlying system). How does this sampling process affect estimates of whether the system is at a critical point? A second difficulty in interpreting these studies stems from the fact that they are based global statistical measures whose relationship with simple statistics such as firing rates and correlations— which are commonly used and have been extensively studied in neural coding [30, 31]—is unclear. We here focus on one statistic that has been used as evidence of critical behaviour, namely the dependence of specific heat on population size and temperature. We study how it depends on neural firing rates and correlations, as well as on how this data is subsampled during data analysis:

First, we show explicitly that signatures of criticality, can be reproduced in canonical feed-forward models of neural population activity, as predicted by previous studies [25, 26, 28]. These studies did not have tools for studying population statistics in large simulations, and they were therefore limited to studying small (*n* ≤ 40) systems– for these small system sizes, it is difficult to make statements about the peak in the specific heat and its scaling with population size. In particular, the dominant peak near unit temperature only emerges for much larger systems. We overcome this difficulty by providing improved algorithms for efficiently fitting maximum entropy models to large neural populations (available at https://github.com/mackelab/CorBinian), and use them to apply the analyses proposed by previous studies [7] to data simulated from a simple, feedforward encoding model of retinal processing [32, 33, 34, 35].

Second, previous theoretical studies [26, 27, 28] treated only the limiting behavior of the specific heat at unit temperature, and did not investigate its dependence on firing rates and correlations. We here relate the characteristic shape of specific heat curves (i.e. the dependence of specific heat on temperature) to neural correlations and firing rates. The emergence of peak specific heat at the ‘inherent’ temperature *T* = 1 has given rise to the idea that correlations in the observed system are ‘special’, i.e. that systems with stronger or weaker correlations would not exhibit them [7]. We use an analytically tractable model of the analysis process to show that this is not the case– the more strongly correlated the population is, the more pronounced signatures of criticality will be. This analysis also shows that a ‘low-temperature’ regime (as reported by [18]) will be found whenever firing rates are sufficiently low.

Third, we analyze the structure of correlations which are sufficient to induce signatures of criticality, and find that it is sufficient if the average correlation is independent of population size. Such ‘criticality-inducing’ correlations can arise both from neural mechanisms such as common input or dense connectivity. Importantly, we show that they can also arise as a consequence of data analysis: Uniformly subsampling a recording with any non-zero correlations to construct subpopulations yields criticality-inducing correlations.

In summary, we show that statements about signatures of criticality derived from thermodynamics can be reduced to statements about firing rates and correlations, and that correlation structures which give rise to these signatures are ubiquitous in neural populations.

## Results

### Signatures of criticality arise in a simple model of retinal ganglion cell activity

**Fig 1:**
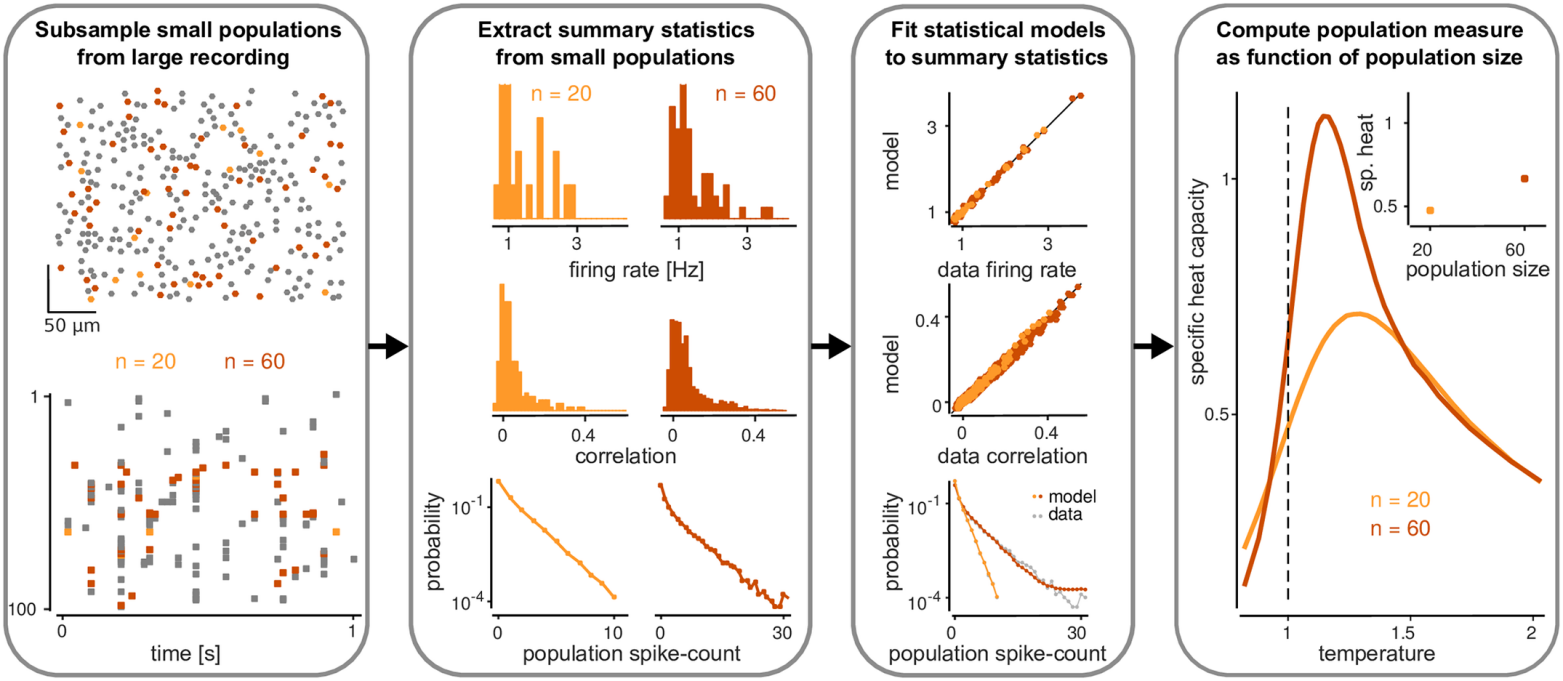
How can one relate theories of thermodynamic criticality to the statistics of neural data? In physical systems, the divergence of specific heat with system size can be interpreted as the system being at a critical point. We here study an analysis approach that has been proposed in order to search for similar signatures of criticality in the statistics of neural population activity. In this approach, different populations are subsampled from a large recording and summary statistics are extracted for each subpopulation (e.g. firing rates, correlations and population spike count statistics). Subsequently, maximum entropy models are fit to these data which assign a probability to each possible spike-pattern. Exploiting the mathematical relationship between the log-variance of probabilities (in statistics) and the specific heat (in thermodynamics) then allows one to compute and study the behaviour of the specific heat with population size. The goal of this study is to determine under which conditions (i.e., for which firing rates and correlations) such an analysis would report that the system is critical. To this end, we apply this approach to a simulation of neural population activity and analytically tractable models.

A hallmark of criticality is that the specific heat capacity of the model diverges when the temperature reaches the critical temperature [5]. Tkačik et al. [7] developed an approach for translating this concept to neural data analysis (see Fig 1):. In this analysis, neural populations of different size *n* are generated from the full recording (of size *N*) by random subsampling. The statistics of activity for each population of size *n* are characterized using a maximum entropy model fit to population activity [13, 14, 16, 17, 3]. Finally, the maximum entropy models are perturbed by introducing a temperature parameter, and specific heat is computed for each population size *n* and temperature *T* from the (perturbed) maximum entropy model fit. Divergence of specific heat with population size *n*, and a peak of the specific heat near unit temperature *T* = 1 (the ‘temperature’ of the original data) are interpreted as indication for the system being at a critical point [7].

**Fig 2:**
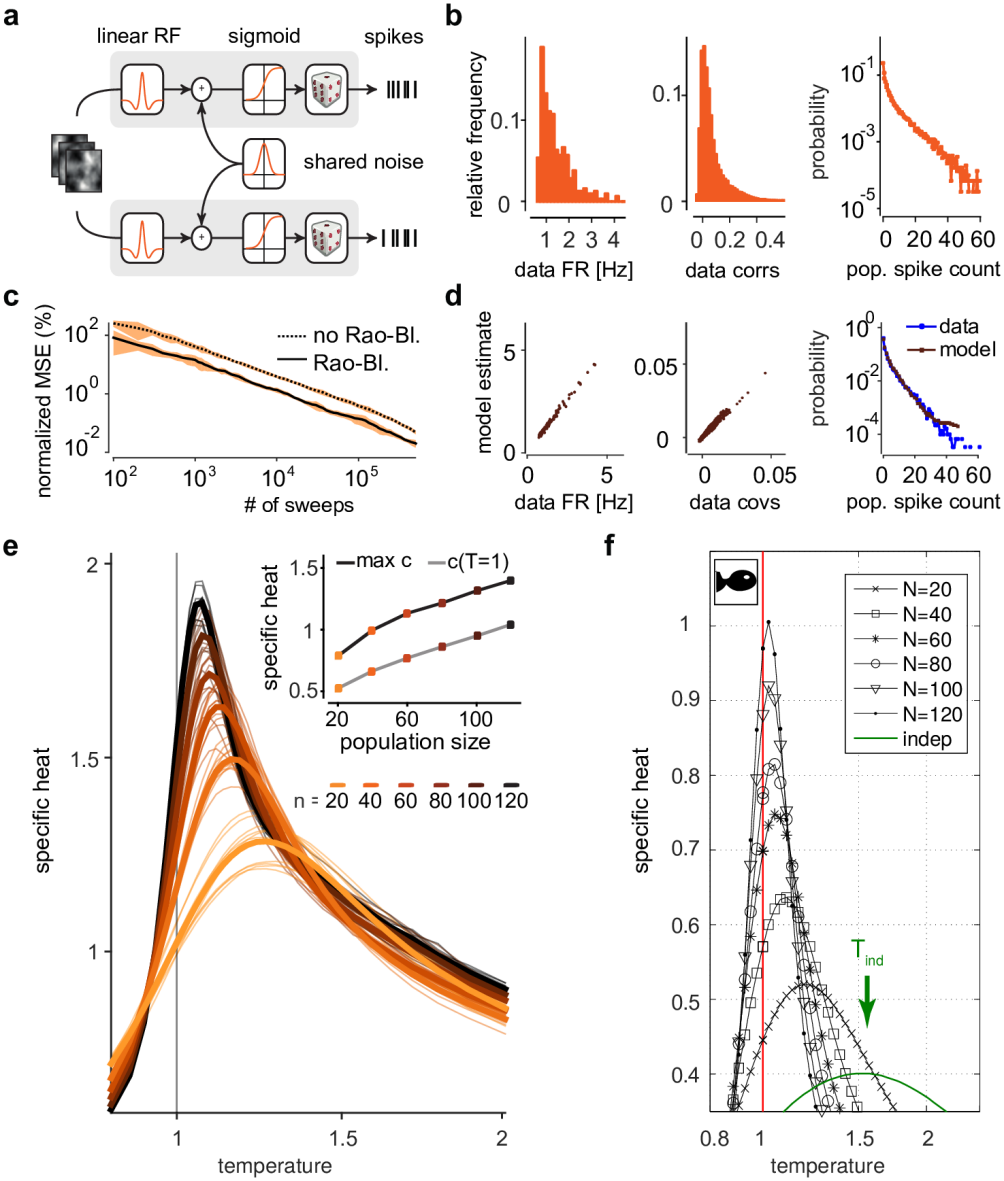
Signatures of criticality in a simulation of retinal ganglion cell activity. **a)** Simulation schematic: Neurons have linear stimulus selectivity with centre-surround receptive fields and correlated Gaussian noise. **b)** Statistics of simulated population activity. Histograms of firing rates (left), correlation coefficients (centre) and frequency of population spike-counts (right). **c)** Estimation-error (normalised mean square error) in pairwise covariances as function of sample size, averaged across 10 populations of size *n* = 100. Rao-Blackwellization reduces the number of samples needed for a given level of accuracy by a factor ≈ 3. **d)** Quality of fit: Population models (here *n* = 100, example population) capture the mean firing rates (left), covariances (centre) and spike-counts (right). **e)** Divergence of specific heat: Average and individual traces for 10 randomly sampled populations for each of 6 different population sizes, exhibiting divergence of specific heat and peak in heat near unit temperature. Inset: Specific heat at unit temperature and at peak vs. population size. **f)** Specific heat for different temperatures and subsampled population sizes (here denoted by capital letter *N*) in recordings of salamander retinal ganglion cells responding to naturalistic stimuli, reproduced from [7].

We wanted to verify that this phenomenon could be captured in feedforward models of retinal processing. We wanted to directly demonstrate that canonical mechanisms of retinal processing—such overlapping centre-surround receptive fields, spiking nonlinearities, shared Gaussian noise—are sufficient for the signatures of criticality to arise. We first created a simple phenomenological model of retinal ganglion cell (RGC) activity based on linear-nonlinear neurons [32, 33, 35]. In this model (Fig 2a), we assumed retinal ganglion cells to have centre-surround receptive fields [36, 35] with linear spatial integration [37], sigmoid nonlinearities and stochastic binary spikes: in each time bin of size 20ms, each neuron *i* either emitted a spike (*x*_*i*_ = 1) or not (*x*_*i*_ = 0). We used a sequence of natural images as stimuli. In addition to the feedforward drive by the stimulus, nearby neurons received shared Gaussian noise, mimicking common input from bipolar cells [30]. Thus, cross-neural correlations in the model arise from correlations in the stimulus, receptive-field overlap and shared noise, but not from lateral connections between RGCs. As we will explain below, only the strength of correlations, but not their mechanistic origin or dependence on stimuli, is relevant for determining the specific heat. Parameters of the model were chosen to approximate the statistics of receptive-field centre locations of RGCs, as well as histograms of firing rates, pairwise correlation-coefficients and population spike-counts (Fig 2b).

We subsampled populations of different sizes 20 ≤ *n* ≤ 120 by uniformly sampling cells from our simulated recording of total size *N* = 316 neurons. For each population we fit a ‘K-pairwise’ maximum entropy model [3]. This model assigns a probability *P*(**x**) to each spike-pattern **x**. It is an extension of pairwise maximum entropy models (i.e. Ising models) [13, 14] which reproduce the firing rates and pairwise covariances, and has additional terms to capture population spike-counts [3] (see Materials for details of model specification and parameterisation). As we needed to efficiently fit this model [38, 39] to multiple simulated data sets, we developed an improved fitting algorithm (see section 1 in S1 Supporting Information) based on maximum-likelihood techniques using Markov chain Monte Carlo (MCMC), building on work by [15]. In particular, we made the most computationally expensive component of the algorithm, the estimation of pairwise covariances via MCMC sampling, more efficient by using a ‘pairwise’ Gibbs-sampling scheme with Rao-Blackwellisation [40] (see section 1.1 in S1 Supporting Information). Most Gibbs-sampling approaches for maximum entropy models [15] update one neuron *i* at a time by re-sampling its state from the conditional distribution, given the state of the other *n* − 1 neurons in the population. We here in each iteration update a randomly chosen pair (*i*, *j*) simultaneously, given the state of the other *n* − 2 neurons. While each pairwise sample is more expensive to compute, this approach has the advantage of yielding a direct estimate of the (conditional) probability of *i* and *j* being active simultaneously. From these conditional probabilities, one can estimate pairwise covariances more efficiently than is possible through averaging samples, a process which is known as Rao-Blackwellization. Here, Rao-Blackwellization resulted in a reduction of the number of samples (and computation time) needed for achieving low-variance estimates of the covariances by a factor of approximately 3 (Fig 2c, Fig. A in S1 Supporting Information). After parameter fitting, the model reproduced the statistics of the simulated data (Fig 2d, Fig. B in S1 Supporting Information).

Following [7], we then introduced a temperature parameter which rescales the probabilities of the model,

$$\begin{array}{c} P_{T}\left(x\right)\propto P{\left(x\right)}^{1/T}, \end{array}$$ (1)

where temperature *T* = 1 corresponds to the statistics of the empirical data. By changing *T* to other parameter values one can perturb the statistics of the system [41]: Increasing temperature leads to models with higher firing rates and weaker correlations (Fig. C in S1 Supporting Information), with *P*_*T*_(**x**) approaching the uniform distribution for large *T*. If the temperature is decreased towards zero, *P*_*T*_(**x**) has most of its probability mass over the most probable spike patterns. We compute the specific heat of a population directly from the probabilistic model fit to data [7], using

$$\begin{array}{c} c\left(T\right)=\frac{1}{n}\text{Var}\left[\log P_{T}\left(X|\lambda \right)\right], \end{array}$$ (2)

i.e. the variance of the log-probabilities of the model with parameters *λ*, normalised by *n*. While specific heat is typically motivated by thermodynamics, in this context it corresponds to a global statistical measure which provides a compact mathematical description of the collective statistical dynamics of the system. Just like the entropy corresponds to the (negative) average log-probability across all population states, the specific heat corresponds to the (normalized) variance of log-probabilities. Thus, specific heat is minimal for data in which all patterns **x** are equally probable, and big for data in which pattern-probabilities span a large range. We used MCMC-sampling to approximate the variance across all probabilities, and used this approach to calculate, for each population of size *n*, the specific heat as a function of temperature (Fig. D in S1 Supporting Information).

We found that the temperature curves obtained from the simulated data qualitatively reproduce the critical features of those that had been observed for large-scale recordings in the salamander [7] and rat [8] retina: The peak of the curves diverges as the population size *n* is increased, and moves closer to unit temperature for increasing *n* (Fig 2e). Consistent with experimental findings [42, 7, 8] (Fig 2f) and [28], we found that specific heat diverged linearly with population size. Finally, and also consistent with experimental studies, the peak specific heat is achieved for *T* > 1, which is what has been interpreted as a ‘low-temperature’ state [18]. These results confirm that signatures of criticality arise in a simple feedforward LN cascade model based on generic properties of retinal ganglion cells, and do not require finely tuned parameters or sophisticated circuitry.

### A tractable mathematical model of the analysis process explains specific-heat curves and low-temperature states

In the phenomenological population model above, we observed that specific heat grew linearly with population size, as it did in previous studies built on experimental data [42, 7, 8, 18]. Different ‘populations’ in these analyses are obtained by subsampling different populations from a large experimental recording, and that the parameters of each of these models are independently fit to each such population. How does this analysis process effect the rate of divergences of the specific heat, and the qualitative shape of specific heat curves? To answer these questions, we build a simple mathematical description of the analysis process: In the original papers, populations of different sizes are obtained by randomly subsampling a large recording (which is itself a sub-sample of the underlying circuit). As the simplest possible description of this sampling process, we assume that there is an underlying, infinitely large neural population, and that each population of size *n* is a random subsample. We assume that the underlying population is homogeneous, i.e. that all neurons have the same mean firing rate and pairwise correlations. As a consequence, K-pairwise maximum entropy models are fully specified by the distribution of population spike-count *K* = ∑_*i*_ *x*_*i*_ [25, 43, 44, 45] for each population of size *n*. We refer to models with this property as ‘flat models’ ([46] calls them ‘reduced’ maximum entropy models).

We introduce a new parametrised flat model in which the spike-count distribution is given by the beta-binomial distribution *P*(*K*|*α*, *β*, *n*), reducing the number of free parameters from *n* to 2. The beta-binomial model is a straightforward extension of an independent (i.e. binomial) population model: At each time-point, a new firing probability *p* is drawn from a beta-distribution with parameters *α* and *β*, and neurons then spike independently with probability *p*. Fluctuations in the latent variable *p* are shared across the population and lead to correlations in neural activity. Therefore, this model is a particular instance of a latent variable model. Signatures of criticality in latent variable models have been studied previously [26, 27, 28]. Our analytically-tractable model provides an explicit construction of how subsampling a large population determines the dependence of specific heat on population size.

**Fig 3:**
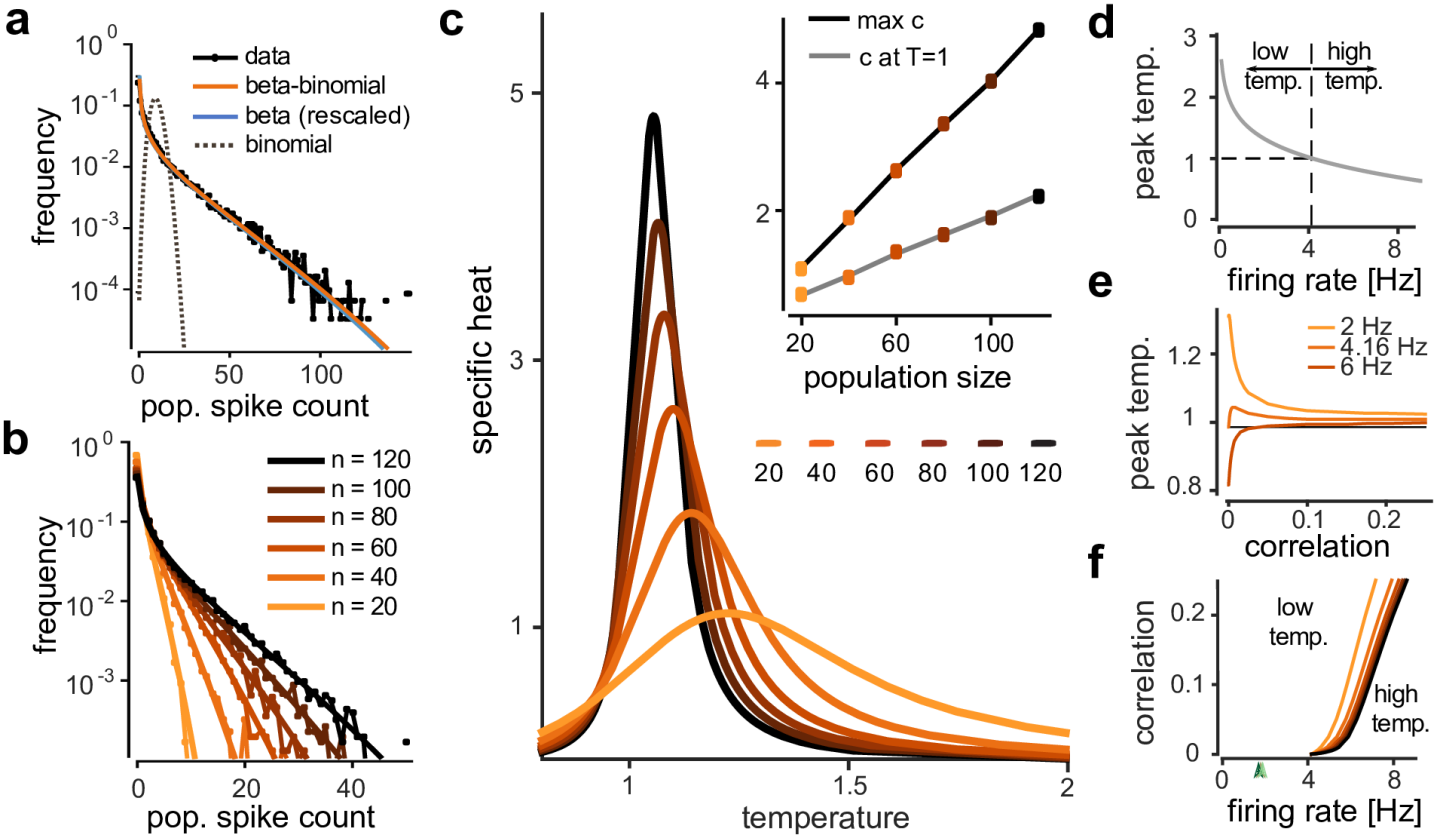
Signatures of criticality and low-temperature states in a mathematically tractable model. **a)** Population spike-count distribution in RGC simulation, and approximation by models. Only the beta-binomial population model fits simulated data accurately, and for the full recording (*N* = 316) closely matches the shape of a beta distribution. **b)** Beta-binomial model fits for different population sizes. **c)** Specific heat traces for beta-binomial model, exhibiting signatures of criticality. Average and individual traces for 30 randomly sampled populations for each of 6 different population sizes. Inset: Specific heat at unit temperature and at peak vs. population size. **d)** Location of peak specific heat for independent model as function of firing rate. For *μ*/Δ = 4.16Hz (assuming Δ = 20ms bins), the peak is above unit temperature, a ‘low-temperature phase’. **d)** Location of peak specific heat as function of correlation, for *n* = 100 and three different firing rates. Peaks cross *T* = 1 only for firing rates ≥ 4.16Hz. **e)** ‘Low’ and ‘high’ temperature phases for beta-binomial model as function of firing rate and correlation strength and for population sizes (*n* = 20 to *n* = 120, colors as in **b**,**c**). Increasing correlations and population size expand the low-temperature regime beyond 4.16Hz. Data sets from previous studies had average firing rates well within low-temperature regime (arrows, colors as in Fig. E in S1 Supporting Information).

Our beta-binomial model provided a good fit to the population spike-count distributions of the simulated data (Fig 3a) across different population sizes *n* (Fig 3b). Importantly, the best-fitting parameters *α* and *β* did not vary systematically across population sizes, and converged to values of *α* = 0.38 and *β* = 12.35 (Fig. E in S1 Supporting Informationa), corresponding to a probability of spiking of *μ* = 0.03 in each bin (i.e. each neuron has an average firing rate of *μ*/Δ = 1.5 Hz) and average pairwise correlations of *ρ* = 0.073. The beta-binomial model also provided good fits to published population spike-count distributions [43, 45, 8], as well as to those of retinal ganglion cell activity under different stimulus conditions in [18] (Fig. E in S1 Supporting Information). When we applied this flat model to populations subsampled from the RGC simulation, we could qualitatively reproduce the specific heat curves of the K-pairwise model (see also Fig. F in S1 Supporting Information). In particular, we found a linearly diverging peak that moved closer to *T* = 1 as the population size was increased (Fig 3c). Thus, linear divergence of specific heat is qualitatively captured by this model of how different populations are obtained by subsampling a large population.

One of the difficulties of interpreting the scaling behaviour of maximum entropy models fit to neural data is the fact that the construction of the limit in *n* differs from those studied in statistical physics: In statistical physics, different ‘*n*’ typically correspond to systems of different total size, and the parameters are scaled as a deterministic function of *n* (e.g. drawn from a Gaussian with variance proportional to 1/*n* in spin-glasses [47, 48]). In studies using maximum entropy models for neural data analysis, populations of different *n* are obtained by randomly subsampling a fixed large recording, and the parameters are fit to each subpopulation individually. Thus, there is no analytical relationship between population size and parameter values in this approach. With our model of the analysis process based on flat models, it is possible to analytically characterise the behaviour of the specific heat for large population sizes for this sampling process [25, 44]. Using this approach, one can show (section 2.3 in S1 Supporting Information and [25] for details) that for virtually all flat models, the specific heat diverges linearly at unit temperature, but not for any other temperature *T* > 1 or *T* < 1 (section 2.4 in S1 Supporting Information). As a consequence, the peak must move to *T* = 1 as *n* is increased. Hence, almost any flat model analysed with the methods developed by [7] will exhibit signatures of criticality. In particular, these results hold also for models which are more weakly or more strongly correlated than real neural populations, and even for models with unrealistic population spike-count distributions (see Fig. G in S1 Supporting Information for an illustration). There are only two exceptions: The first one is a model in which all neurons are independent (i.e. a binomial population model), and the second one is a flat pairwise maximum entropy model—indeed, this is the only flat model with non-vanishing correlations for which the specific heat does not have its peak at unit temperature (see [25] for an illustration for the flat pairwise maximum entropy model).

Finally, it has been observed that the peak of the specific heat curve is consistently ‘to the right’ of *T* = 1, which was interpreted as the neural population activity in the retina being in a ‘low-temperature state’ [18]. Our analysis based on the flat model gives insights into this phenomenon: For correlation *ρ* = 0, the position of the peak can be calculated in closed form (Fig 3d). We observe that the peak will be at temperatures >1 whenever the spike probability is smaller than μ* = 0.0832, which corresponds to a firing rate of μ*/Δ = 4.16Hz at a bin size of Δ = 20ms. Thus, in our model, the ‘temperature-state’ of a population can be reduced to a statement about the firing rate relative to the bin size used for analysis: For *ρ* > 0 (Fig 3e) and for larger population sizes *n*, the firing rate at which the transition occurs are shifted to slightly higher firing rates, i.e. the ‘low-temperature’ regime is even bigger, and e.g. extends to firing rates up to 8.63Hz for average correlations of *ρ* = 0.25 and population size *n* = 120 (Fig 3f). While this dependence may be more complicated for full correlation structures, our analysis again connects global population measures from statistical mechanics to basic, directly measurable statistics of neural data: ‘being in a low-temperature state’ is a statement about the firing rates in the population being low.

### Strong neural correlations lead to fast divergence of specific heat

**Fig 4:**
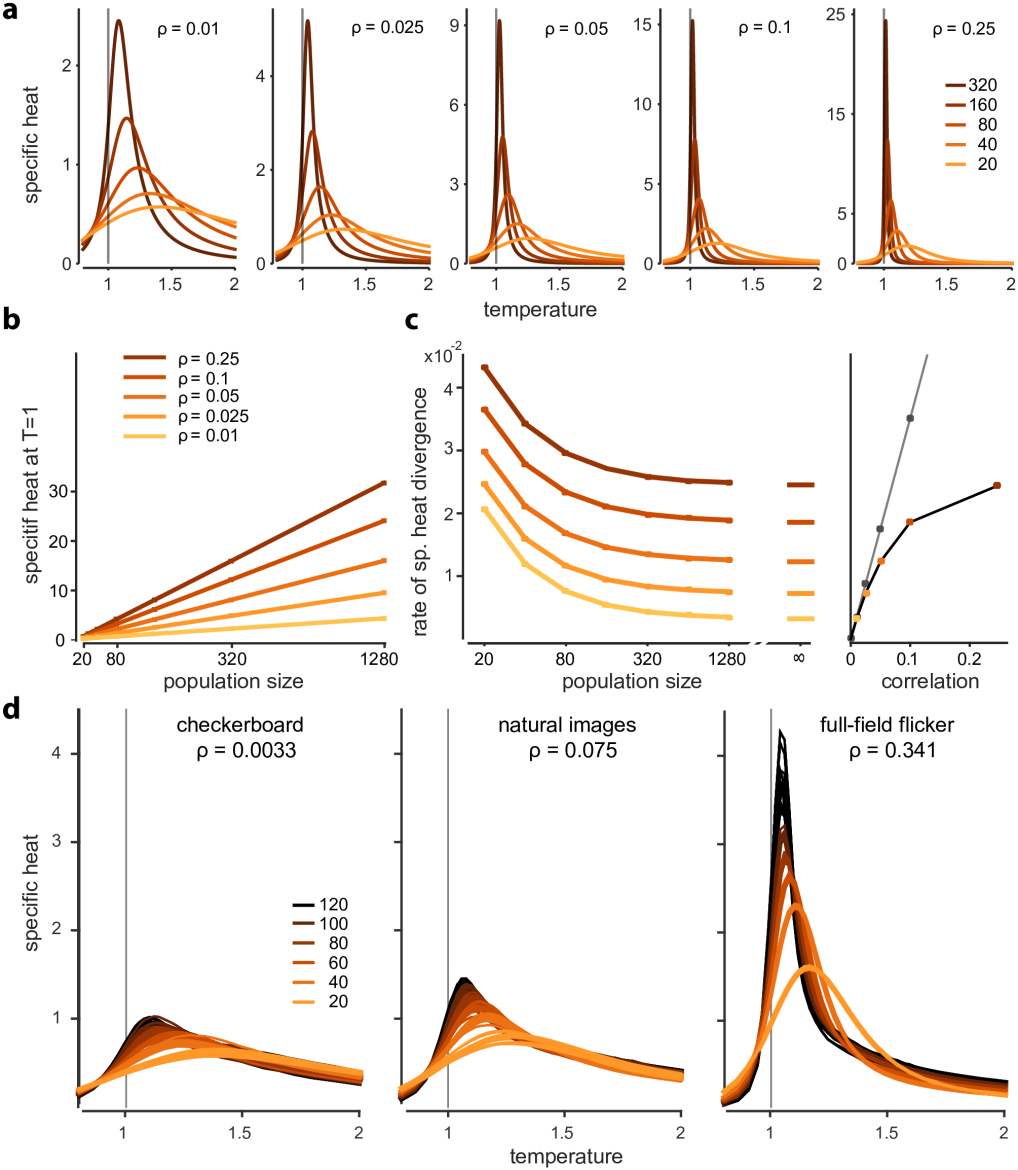
Relationship between correlations and criticality. **a)** Specific heat traces for beta-binomial model, different correlation strengths and population sizes. Heat traces are qualitatively similar, but differ markedly quantitatively (see y-axes). **b)** Specific heat diverges linearly, and the slope depends on the strength of correlations. **c)** Divergence rate of specific heat for beta-binomial model as a function of correlation strength (left). Rightmost point (at infinity) corresponds to analytical prediction of large-*n* behaviour. Divergence rates are strictly increasing with correlation strength (right) which is captured by a weak-correlation approximation (dashed line). **d)** Specific heat increases with correlation in the K-pairwise maximum entropy model: average and individual traces for 10 randomly subsampled populations for 6 different population sizes. Left to right: checkerboard, natural images and full-field flicker stimuli presented to the population. Correlation strengths denote mean correlation coefficient in each population.

The rate at which the specific heat diverges provides a mean of quantifying the ‘strength’ of criticality. What is the relationship between correlations in a neural population and the rate of divergence? To study how the specific heat rate $\tilde{c}=c\left(T=1\right)/n$ depends on the strength of correlations, we used a beta-binomial model to generate simulated data with firing rate *μ*/Δ = 1.5Hz (i.e. each neuron has a probability of spiking of *μ* = 0.03 per bin), and different pairwise correlation coefficient *ρ* ranging from *ρ* = 0.01 to *ρ* = 0.25 (Fig 4a). The heat curves had the same shape as in the analyses above, with a peak that increases and moves to unit temperature (Fig 4b). We found that the specific heat rates $\tilde{c}$ increased strictly monotonically with *ρ* (Fig 4b and 4c). For the beta-binomial model, the large-n value of $\tilde{c}$ can be calculated analytically (section 3.2 in S1 Supporting Information for details) as a function of the parameters *α* and *β*,

$$\begin{array}{ll} \tilde{c} & =\frac{\alpha \left(\alpha +1\right)\psi_{1}\left(\alpha +1\right)+\beta \left(\beta +1\right)\psi_{1}\left(\beta +1\right)}{\left(\alpha +\beta \right)\left(\alpha +\beta +1\right)}\\ & +\frac{\alpha \beta {\left(\psi_{0}\left(\alpha +1\right)-\psi_{0}\left(\beta +1\right)\right)}^{2}}{{\left(\alpha +\beta \right)}^{2}\left(\alpha +\beta +1\right)}-\psi_{1}\left(\alpha +\beta +1\right), \end{array}$$ (3)

where *ψ*_0_, *ψ*_1_ denote the di- and trigamma function, respectively. This analytical evaluation of $\tilde{c}$ (valid for large *n*) was in good agreement with numerical simulations (Fig 4c left). In the case of weak correlations *ρ*, eq 3 can be simplified: In this case, the specific heat rate is proportional to the strength of correlations (section 3.1 in S1 Supporting Information for details), i.e.

$$\begin{array}{c} \tilde{c}\approx \rho \;\mu \left(1-\mu \right){\left(\text{log}\left(\frac{1-\mu }{\mu }\right)\right)}^{2}, \end{array}$$ (4)

and also increases strongly with firing rate for small *μ* (Fig. H in S1 Supporting Information). This expression can also be derived from the Gaussian model in [8] equation (4), by inserting the expected values of the mean and variance of the population spike-count under random subsampling. The monotonic relationship between correlations and specific heat is also consistent with the derivation in [27] for latent-variable models: inspection of equation (65) in [27] shows that the specific heat is related to a sum of conditional entropies– for binary random variables, these entropies are monotonically related to covariances, which effectively shows that, in their model, specific heat also increases with correlations.

We found that the relationship between the strength of correlations and the ‘strength’ of criticality (i.e. the divergence rate of specific heat) also held in simulations of feedforward models of retinal population activity. In the original study [7], specific heat was computed from K-pairwise model fits to RGC activity resulting from three different kind of stimuli: random checkerboard stimuli (which do not have long-range spatial correlations, although stimulus-driven cross-neural correlations can arise from receptive field overlap), natural stimuli, which exhibit strong spatial correlations, and full-field flicker (which constitutes an extreme case of spatial correlations since all pixels in the display are identical). It was found that specific heat diverges in all three conditions (consistent with a more recent study [18]), and interpreted this as evidence that signatures of criticality are not ‘inherited from the stimulus’ [7]. When we simulated responses to different stimuli we found the divergence rates of the specific heat to follow the pattern of induced correlation strength, consistent with the monotonic relationship between correlation strength and specific heat growth rate shown above for the flat models (Fig 4d): For populations size *n* = 100, checkerboard/natural/full-field flicker stimulation lead to average correlation strengths of *ρ* = 0.033/0.075/0.341, respectively, and to specific heat growth rates of $\tilde{c}=0.0029/0.0046/0.0104$.

Tkačik et al. had found the lowest peak in divergence rate for checkerboard (max *c* ≈ 0.54), higher peak-divergence rates for natural movies (max *c* ≈ 0.92) and the highest peak for full-field flicker (max *c* ≈ 2.4, all results for *n* = 100). Thus, the ordering of the peak values of specific heat in their study is consistent with our results. However, when comparing the values at *T* = 1, they found a slightly higher divergence rate for natural movies ($\tilde{c}\approx 0.005$) than for full-field flicker ($\tilde{c}\approx 0.004$). This mismatch could result from adaptation or temporal dynamics of the stimulus affecting firing rates or correlations in their data [20], or from our simulations not precisely matching the statistics of their experimental data.

These statements also qualitatively hold in a modified temperature analysis [7] in which firing rates are kept constant (at the firing rates of *T* = 1) when temperature is varied (section 3.4 in S1 Supporting Information and in Fig. I in S1 Supporting Information). We conclude that the experimental evidence—which showed that the specific heat diverges, and how the speed of divergences depends on the stimulus ensemble—is largely consistent with a simple, feedforward phenomenological model of retinal processing. Thus, at least for flat models, ‘being very critical’ is a consequence of ‘being strongly correlated’, and not evidence for correlations being fine-tuned or self-organized to a particular value.

### Random subsampling gives rise to criticality-inducing correlations

**Fig 5:**
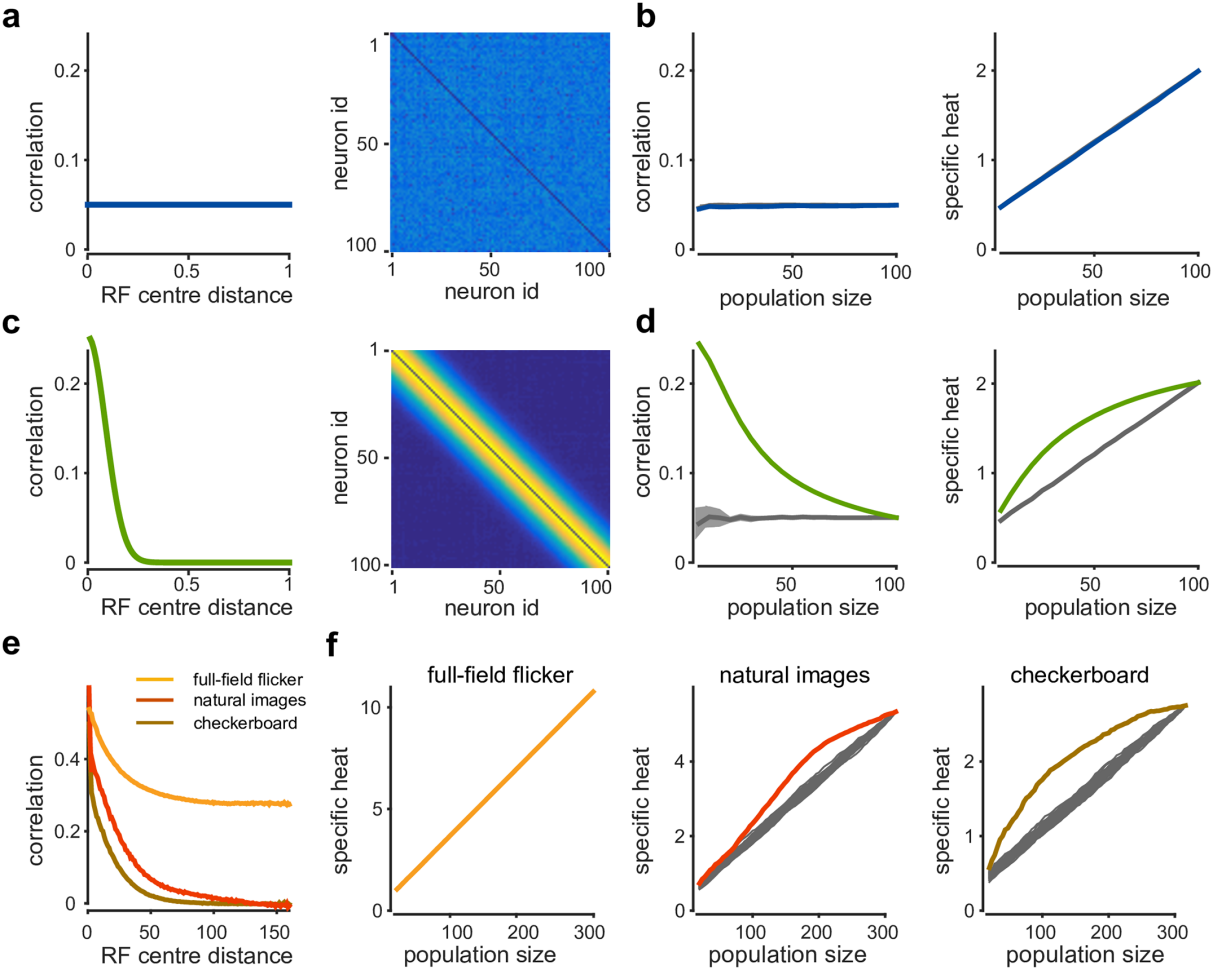
Random subsampling leads to criticality-inducing correlations. **a)** Illustration: A population with 100 neurons and infinite-range correlations, the average correlation between any pair of neurons is close to 0.05. Correlation as function of inter-neuron distance (left) and full correlation matrix (right). **b)** Average correlation in subpopulation of different size *n* (left) and specific heat at *T* = 1 as function of *n* (right), when neurons are sampled from 1 to 100 (blue). Random sampling gives identical results (gray). **c)** Population with limited-range correlations, same plots as in panel a. **d)** Left: Average correlation as function of population size for spatially structured sampling (green) and uniform subsampling (gray). Right: Specific heat at *T* = 1 grows linearly for random subsampling, but shows signs of saturation for spatially structured sampling. **e)** Average correlation as function of inter-neuron distance in RGC simulation. For checkerboard and natural images, correlations drop to 0 for large distances. **f)** Specific heat at *T* = 1 for different stimulation conditions, for spatially structured (colour) or random subsampling (gray).

In the above, we showed that a beta-binomial spike-count distribution can be sufficient for signatures of criticality to arise. For this to hold we need the variance of the population spike-count to grow quadratically with population size, i.e. Var(*K*) ∝ *n*^2^. The variance of the population spike-count is equal to the sum of all variances and covariances in the population, $\text{Var}\left(K\right)=\sum_{i=1}^{n}\text{Var}\left(x_{i}\right)+\sum_{i\neq j}\text{Cov}\left(x_{i},x_{j}\right)$. A sufficient condition for signatures of criticality to arise in these models is that the average covariances (and hence correlations) between neurons are independent of *n*, $\frac{1}{n(n-1)}\sum_{i\neq j}\text{Cov}\left(x_{i},x_{j}\right)\approx \text{constant}$ [27, 6, 5]. We refer to correlations with this property as ‘criticality inducing’. One possible criticality-inducing correlation structure are so called ‘infinite range’ correlations: correlation between neurons do not drop off to zero for large spatial distances. In the extreme case of distance-independent correlations (Fig 5a), adding more and more neurons to a population will not change the average pairwise correlation within the population (Fig 5b). We note that infinite-range correlations are typically not present in the thermodynamic limit in physical systems at equilibrium. In neural systems, infinite-range correlations could be a consequence of densely connected circuitry, or of a shared stimulus drive.

Importantly, criticality-inducing correlations can also result as a consequence of subsampling a large neural population: Even a neural population which does not have infinite-range correlations can appear critical if it is randomly subsampled during analysis. If different populations of size *n* are obtained as above by (uniformly) subsampling a large recording of size *N*, then the pairwise correlations in each subpopulation are also a random subsample of the large correlation matrix of the full recording. For any correlation structure on the full recording (including limited-range correlations, Fig 5c), the expected average correlation in a population of size *n* is identical to the average correlation in the full recording and hence independent of *n* (Fig 5d left, grey line). Despite the pairwise correlations being subsampled in blocks of principal submatrices rather than independently, the variance of the average correlation can drop with the square of the population size *n*, and is guaranteed to fall at least as 1/*n* (section 4.1 in S1 Supporting Information, and Fig. J in S1 Supporting Information). Because the average correlation will be independent of *n* and have negligible variance (Fig 5d left, shaded area), specific heat will diverge with constant slope (Fig 5d right). In contrast, if different population sizes are constructed by taking into account the spatial structure of the population (i.e. by iteratively adding neighbouring cells) then the average correlation in each subpopulation will drop with *n*, and the slope of specific heat growth will decrease with population size.

In our RGC simulation, pairwise correlations did drop off to zero with spatial distance for checkerboard and natural images, but not for full-field flicker (Fig 5e). Pairwise correlations in the full-field flicker condition initially drop off due to distance-dependent shared noise, but eventually saturate at a level far above zero that is determined by the full-field stimulus. Due to these strong infinite-range correlations, both spatially structured sampling and uniform sampling then give rise to linear growth in specific heat (Fig 5f left). For the other two stimulus conditions, however, the choice of subsampling scheme does result in markedly different behavior of the specific heat growth: Both for natural images and checkerboard stimuli, we can see the rate of growth decreases for large *n* under spatially structured subsampling (Fig 5f centre and right). This effect will be more pronounced for larger simulations, and in additional simulations we found specific heat to saturate once populations are substantially bigger than the spatial range of correlations. This behavior is not unique to the simplified flat models. Specific heat traces computed from K-pairwise models fit to populations obtained with spatially structured sampling also show a marked decrease in specific heat growth rates (section 4.2 in S1 Supporting Information and Fig. K in S1 Supporting Information).

In summary, populations will exhibit critical behaviour if correlations have infinite range (over the size of the recording), irrespective of the sampling scheme. In addition, if a population is randomly subsampled (as was done in [7, 8]), then signatures of criticality will arise even if the underlying correlations have limited range.

## Discussion

An intriguing hypothesis about the collective activity of large neural populations has been the idea that their statistics resemble those of physical systems at a critical point. In recent years, several studies [12, 5, 6, 11, 7, 8, 18] proposed a new approach to studying criticality in biological data, motivated by notions of criticality in thermodynamics. Signatures of criticality have also been observed in natural images [11] and cortical populations [6], and have been studied using the theory of finite-size scaling and critical exponents [6]. It has been argued that systems close to a critical point might be optimally sensitive to external perturbations [6] and that the large dynamic range of the code (i.e. large variance of log-probabilities) might be beneficial for encoding sensory events which likewise have a large distribution of occurrence probabilities [16].

This hypothesis that neural systems are poised at a thermodynamic critical point could open up further questions on how the system maintains its critical state and on implications for how neural populations encode sensory information and perform computations on it. Alternatively, generic mechanisms could be sufficient to give rise to data which satisfies the definition of criticality put forward in these studies. We had demonstrated in a previous theoretical study [25] that simple models with Gaussian common input can exhibit a diverging specific heat. More recently, it was shown [26, 27, 28] that common input (or other latent variables which lead to shared modulations in firing rates, such as non-stationarity [29]) can give rise to Zipf-like scaling of pattern probabilities, a second signature of criticality. Mathematically, Zipf’s Law is equivalent to stating that the plot of entropy vs energy (i.e. log-probability) is a straight line with unit slope [26, 27]. Schwab et al [26] showed that particular latent variable models give rise to Zipf’s law. This result was generalized [27, 28] to show that, under fairly general circumstances, high-dimensional latent variable models exhibit a wide distribution of energies (i.e. log-probabilities) and hence a large specific heat. It has also been argued that the use of data sets which are too small might give rise to spuriously big specific heats [49]: while this could be true in principle, additional analyses e.g. in [7] show that their results are robust with respect to data set size, and our results are also valid even in the case of infinite data. Finally, it has also been suggested that whether statistical models exhibit criticality depends on which variables are measured and constrained by the model fit [50, 51].

Previously, criticality in neural systems has also been investigated extensively using a definition of criticality which is based on temporal dynamics with power-law statistics, so-called ‘avalanches’ [52, 5]. Numerous studies have reported and studied ‘avalanche criticality’ [8, 21], proposed possible mechanisms (e.g. based on self-organization [53]), and discussed finite-size effects and sub-sampling [54], as well as a need for rigorous statistical analysis [55]. We emphasize that the ‘avalanche’ definition of criticality is not equivalent to the thermodynamics-inspired definition used in these more recent studies [12, 8]. Our study is only concerned with this more recent approach, and our results thus have no bearing on studies of ‘avalanche-criticality’.

We here related signatures of criticality to the structure of firing rates and correlations in the population: We found that average correlations which are independent of population size are sufficient for inducing criticality, irrespective of their origin. In the thermodynamic analysis of physical systems at equilibrium, long-range correlations typically vanish in the thermodynamic limit. In neural systems, however, ‘criticality-inducing’ correlations can arise as a consequence of various factors: First, in a local patch of retina, retinal ganglion cells have a large degree of receptive field overlap, and natural stimuli also contain strong spatial correlations. This can lead to correlations which do have unlimited range within the experimentally accessible length scales. Thus, fluctuations in the stimulus will lead to common activity modulations amongst neurons within the population. Empirically, correlations between pairs of retinal ganglion cells only fall off slowly with the distance between somata (or receptive field centres) [35]. Second, firing rates e.g. of cortical neurons are modulated by global fluctuations in excitability [45, 56], resulting in neural correlations with infinite range. Third, and importantly, we showed that criticality-inducing correlations can also arise as a consequence of data analysis choices: Uniformly subsampling a large recording with correlations to construct subpopulations yields criticality-inducing correlations, even if the correlations itself do not have unlimited range.

We also showed that there is a direct relationship between ‘how critical’ and ‘how correlated’ a population is: The stronger correlations are, the more prominent the divergence in specific heat is. Mechanisms underlying correlations in spiking activity have been extensively studied in neuroscience [30, 31], and our study makes it possible to relate ‘signatures of criticality’ derived from thermodynamics to these studies, and to interpret the significance of observing these effects: Given the ubiquity of criticality-inducing correlations, signatures of criticality are likely going to be found not just in retinal ganglion cells, but in multiple brain areas and model systems. They are entirely consistent with canonical properties of neural population activity, and require neither finely-tuned parameters in the population, nor sophisticated circuitry or active mechanisms for keeping the system at the critical point. The relationship between firing rates, correlations and criticality (eqs 3 and 4) also yields a prediction about how adaptation in a classical sense should modulate signatures of criticality: The height of the peak is monotonically related to both correlation strength and firing rate. Adaptation typically reduces firing rates and correlations [57, 58]. Taken together, this leads to the prediction that adaptation should *reduce* signatures of criticality– this is precisely the opposite of what has been predicted in [7]. Finally, the dependence of specific heat on correlations might also be an explanation of why Ioffe and Berry [18] found that a feedforward model fit to their retinal data (which had lower correlations) underestimated the specific heat.

In summary, we conclude that current attempts to interpret findings of thermodynamic criticality in neural population activity have limited potential to lead to new insights into theories of neural computation– in particular, they are not able to discriminate between different hypotheses about either the origin or the functional consequence of the statistics of neural activity. A reliable interpretation of any test for criticality is possible only in reference to a-priori knowledge about the outcome of the test on relevant ground truth models. In order to realise the potential of large-scale recordings of neural activity in the search of a theory of neural computation, we will need data analysis methods which are adapted to the specific properties of biological data, and in particular the fact that neural activity is highly subsampled [59, 60, 54, 61]. One approach to dealing with subsampled data is to use latent-variable models which explicitly model the effect of unobserved inputs and states [62, 63]. In addition, we will also require hypotheses about the normative principles which govern their computations. A possible link between neural activity and theories of criticality might emerge from recent work in machine learning, which is starting to study links between the information-processing capabilities of artificial neural networks and critical phenomena [64].

## Materials and methods

### Retina simulation

We simulated a population of *N* = 316 retinal ganglion cells as linear threshold neurons whose receptive fields were modelled by difference-of-Gaussian filters with ON-centres [37, 35, 33]. The simulation comprised two subgroups of cells with different receptive field sizes (surrounds 56μm and 30μm in retinal space, centres 28μm and 15μm, respectively, one third cells with large receptive fields). For both subgroups, the weight of the surround was 0.5 of the centre weight. Locations of receptive field centres (Fig 1 left panel) were based on a reconstruction of 518 soma locations from a patch of mouse retina [65]. As the reconstructed locations in that data set also comprised about 40% amacrine cell somata, we randomly discarded 40% of the cell locations. The resulting patch of retina covered an area of 200 × 300μm^2^, corresponding to 100 × 150 pixels in stimulus space. Correlated noise across neurons was modelled using correlated additive Gaussian noise. Correlations dropped off exponentially with soma distance with a decay constant of *τ* = 30μm i.e. noise covariance matrix was chosen as $\Sigma =\sigma_{noise}^{2}\left(aI_{n}+be^{-D/\tau }\right)$, where *D*_*ij*_ is the distance between neurons *i* and *j* and *a*^2^ + *b*^2^ = 1. We set *σ*_*noise*_ = 0.022 and *a* = 0.45. We modelled neural spiking in discrete time using 20ms bins. In each bin *t*, the total input *z*_*i*_(*t*) to neuron *i* was given by $z_{i}\left(t\right)=w_{i}^{⊤}s\left(t\right)+\epsilon_{i}\left(t\right)$, where *w*_*i*_ is the receptive field of neuron *i*, *s*(*t*) the vectorised stimulus and *ϵ*_*i*_(*t*) the input noise of neuron *i*. A neuron in a given bin is active (*x*_*i*_ = 1) if *z*_*i*_ + *d* > 0.5 and inactive (*x*_*i*_ = 0) otherwise, with offset *d* = 0.168 [66]. Parameters of the simulation (centre and surround sizes, relative strength of centre and surround, magnitude and correlations of noise, spiking threshold) were chosen to roughly match the statistics of neural spiking (firing rates, pairwise correlations, population activity counts) reported in studies of salamander retinal ganglion cells [13, 3, 2].

### Stimuli

We used three types of stimuli for this study: natural images, checkerboard patterns and full-field flicker. For natural image stimuli, we used a sequence of 101 images of foliages. Each image was 400 × 400 pixels, and each image was presented for 20ms with 300 repetitions total. The luminance histograms of the images were transformed to a normal distribution with mean 0.5 and pixel values between 0 and 1.

For the full-field flicker stimulus, luminance levels were drawn from a Gaussian distribution with mean *μ* = 0.5 and variance *σ*^2^ = 0.06. Checkerboard stimuli consisted of 80 × 80 tiles of size 5 × 5 pixels each. Luminance levels (from within the interval [0, 1]) of each tile were chosen to be either 0.15 or 0.77 with probability 0.5. The parameters of both stimulus sets were chosen to match the dynamic range of the simulated retinal ganglion cells. For both types of stimuli, 2000 images were generated and the image sequences were presented with 10 repetitions. To calculate specific heat as function of increasing population size, we randomly selected 10 subsamples of the full simulated population of *N* = 316 cells at population sizes *n* ∈ {20, 40, 60, 80, 100, 120} by uniformly drawing *n* neurons out of the full population without replacement.

### Statistical model

We modelled retinal ganglion cell activity by using a ‘K-pairwise’ maximum entropy model [3]. In a maximum entropy model [67], the probability of observing the binary spike word **x** ∈ {0, 1}^*n*^ for parameters *λ* = {*h*, *J*, *V*} is given by

$$\begin{array}{c} P\left(x|\lambda \right)=\frac{1}{Z(\lambda )}\exp(h^{⊤}x+x^{⊤}Jx+\sum_{k=0}^{n}V_{k}\delta (K(x)=k)) \end{array}$$ (5)

Here, the parameter vector *h* (of size *n* × 1) and the upper-triangular matrix $J\in R^{n\times n}$ correspond to the bias terms and interaction terms in a pairwise maximum entropy model (also known as an Ising model or spin-glass) [13]. The term $K\left(x\right)=\sum_{i=1}^{n}x_{i}$ denotes the population spike-count, i.e. the total number of spikes across the population within a single time bin, and the indicator-term *δ*(*K* = *k*) is 1 whenever the population spike-count equals *k*, and is 0 otherwise. The term $\sum_{k=0}^{n}V_{k}\delta (K=k)$ was introduced [3] to ensure that the model precisely captures the population spike-count distribution of the data using *n* additional free parameters. The partition function *Z*(λ) is chosen such that the probabilities of the model sum to 1.

### Parameter fitting

To fit the model parameters λ = {*h*, *J*, *V*} to a data set, we maximised the penalised log-likelihood [68, 69] of the data $D=\{x^{\left(1\right)},x^{\left(2\right)},\ldots ,x^{\left(M\right)}\}$ under the model,

$$\begin{array}{ll} L\left(h,J,V\right): & =\sum_{m=1}^{M}\text{log}P(x^{\left(m\right)}|h,J,V)\\ & -\frac{1}{\sigma_{h}}\|h{\|}_{1}-\frac{1}{\sigma_{J}}\|J{\|}_{1}-\frac{1}{2}V^{T}\Sigma^{-1}V. \end{array}$$ (6)

Here, the *l*1-penalty controlled the magnitudes of parameters *h*, *J*, the term ‖*J*‖_1_ favoured sparse coupling matrices, and the regularisation term *Σ* on the *V*-parameters ensures that the terms controlling the spike-count distribution vary smoothly in *k* (section 1 in S1 Supporting Information). This smoothness prior is particularly important for large spike counts, as it makes it possible to interpolate parameters for which the number of observed counts is small.

In maximum entropy models, exact evaluation of the penalised log-likelihood and its gradients requires the calculation of expectations under the model, E[*x*_*i*_], E[*x*_*i*_ *x*_*j*_] or equivalently *cov*(*x*_*i*_, *x*_*j*_), and *P*(*K* = *k*) (section 1.1 in S1 Supporting Information), which in turn requires summations over all 2^n^ possible states **x** and is prohibitive for *n* > 20. Following previous work [15], we used Gibbs sampling to approximate the relevant expectations (section 1.1 in S1 Supporting Information for derivations and implementation details). We used two modifications over previous applications of Gibbs sampling to fitting maximum entropy models to neural population spike train data, with the goals of speeding up parameter learning and alleviating memory usage:

First, we use Rao-Blackwellisation [40] to speed up convergence of the estimation of covariances of **x**: for this, we used pairwise Gibbs sampling (blocked Gibbs with block size 2), where each new sample in the MCMC chain was obtained by updating two entries *i* and *j* of **x** at a time, rather than just a single entry. This allowed us to get estimates of the conditional probabilities *P*(*x*_*i*_ *x*_*j*_ = 1|*x*_∼{*i*,*j*}_), and to use them to speed up the estimation of the second moment *E*[*x*_*i*_ *x*_*j*_] from empirical average of these conditional probabilities (section 1.1 in S1 Supporting Information).

Second, we used a variant of coordinate ascent that calculated all relevant quantities as running averages over the MCMC sample, and thereby avoided having to store the entire $n\times \tilde{M}$ MCMC sample in memory [15], where $\tilde{M}$ is the length of the sample. Because all features of the maximum entropy model are either 0 or 1 (*x*_*i*_, *x*_*i*_ *x*_*j*_ and the indicator function for the spike count), the gain in log-likelihood obtainable from either updating a single element of *h* or *J* [15, 39], or from updating all *V* simultaneously (but not from updating multiple entries of *h* and *J*) can be computed directly from MCMC estimates of E[*x*_*i*_], E[*x*_*i*_ *x*_*j*_] and *P*(*K* = *k*) (section 1.2 in S1 Supporting Information). For each iteration, we calculated the gain in log-likelihood for each possible update of *h*_*i*_, *J*_*ij*_ and full *V*, and picked the update which led to the largest gain [15].

We measured the length of Markov chains in sweeps, where one sweep corresponds to one round of *n*(*n* − 1)/2 Markov chain updates that encompasses all pairs of entries of **x** in random order. We set a learning schedule that started at 800 sweeps for the first parameter update and doubled the number of sweeps in the chain after each set of 1000 parameter updates. We monitored convergence of the algorithm using a normalised mean square error between empirical E[*x*_*i*_], *cov*(*x*_*i*_, *x*_*j*_), *P*(*K* = *k*) and their estimates from the MCMC sample. For normalisation, we used the average squared values of the target quantity, e.g. $\frac{1}{n}\overset{n}{\underset{i=1}{\sum }}\text{E}{\left[x_{i}\right]}^{2}$ for the firing rates. We stopped the algorithm when a pre-set threshold was reached (0.01%, 0.25%, 0.01% for *E*[*x*_*i*_], *cov*(*x*_*i*_, *x*_*j*_), *P*(*K* = *k*), respectively), or when the fitting algorithm took more than ${\left(\frac{n}{100}\right)}^{2}\times 72\text{h}$ of computation time on a single core (2.294 GHz AMD Opteron(TM) Processor 6276) (Fig. A in S1 Supporting Information). For 10 populations of size *n* = 100 (for natural images), the normalised MSEs after model-fitting were 0.43%, 2.80%, 0.42%). An implementation of the fitting algorithms in MATLAB is available at https://github.com/mackelab/CorBinian.

### Specific heat calculation

To investigate thermodynamic properties of neural population codes, Tkačik et al [7] introduced a temperature parameter *T* for eq 5:

$$\begin{array}{c} P_{T}(x|\lambda )=\frac{1}{Z_{T}}\exp(\frac{1}{T}(h^{⊤}x+x^{⊤}Jx+\sum_{k=0}^{n}V_{k}\delta (K(x)=k))) \end{array}$$ (7)

Model fits are obtained at *T* = 1, and the temperature parameter *T* is scaled to study the system (i.e. characterised by *P*_*T*_(**x**|*h*, *J*, *V*) for *T* = 1). Varying *T*, in effect, modulates probabilities by exponentiating them with 1/*T*,

$$\begin{array}{c} P_{T}\left(x\right)\propto {\left(P_{T=1}\left(x\right)\right)}^{1/T}, \end{array}$$ (8)

and that the family of probability distributions obtained by varying *T* can be constructed for any distribution, not just maximum entropy models. For large temperatures *P*_*T*_ approaches a uniform distribution (*P*_*T*_(**x**) ≈ 2^−*n*^ for each **x**), whereas for small temperatures it converges to a singleton, *P*_*T*_(x*) ≈ 1 with x* = *argmax*_**x**_(*P*_*T* = 1_(**x**)).

The specific heat, as given in eq 2, can be obtained from the variance of the log-probabilities of the model. As the variance in practice cannot be computed for large *n*, we obtained estimates of *c*(*T*) using a pairwise Gibbs sampler. The specific heat does not depend on *Z*_*T*_, as changing *Z*_*T*_ results in a constant, additive shift in log-probabilities which does not affect the variance. We tracked the variance of log-probabilities over an MCMC chain $x^{\left(1\right)},\ldots ,x^{\left(\tilde{M}\right)}$ of length $\tilde{M}$ sampled at temperature *T*, using

$$c\left(T\right)\approx \frac{1}{n}\left(\hat{\text{E}}\left[\text{log}P_{T}{\left(x^{\left(m\right)}|\lambda \right)}^{2}\right]-\hat{\text{E}}{\left[\text{log}P_{T}\left(x^{\left(m\right)}|\lambda \right)\right]}^{2}\right)$$ (9)

where $\hat{\text{E}}$ denotes the average over spike words **x**^(*m*)^ sampled from the the MCMC chain. For each population, we evaluated *c*(*T*) for 31 temperatures between *T* = 0.8 and *T* = 2, and found the Gibbs sampler to provide reliable estimates over this temperature range—we in particular chose the minimal temperature *T* = 0.8 larger than previous previously in [7] to minimize possible effects from the sampler getting stuck (see e.g. [46]). We used a burn-in of 2.0e4 sweeps, and ran the sampler for ${\left(\frac{n}{100}\right)}^{2}\times 4\text{h}$ of CPU time, resulting in between 9.97e5 and 1.72e6 sweeps for *n* = 100 (i.e. between 4.94e9 and 8.52e9 sampled individual spike words).

### Simplified population models

For the theoretical analysis of the sampling process, we adopted a class of population models (here referred to as ‘flat’ models) in which all neurons are drawn from an infinite pool of neurons which all have identical mean firing rates, pairwise correlations and higher-order correlations [44, 25, 70, 3, 71]. Such a model is fully specified by the population spike-count distribution *P*(*K* = *k*), and all spike words with the same spike count are equally probable. As a result, the probabilities of individual patterns **x** can be read off from the spike-count distribution by

$$P\left(x\right)={\left({}_{k}^{n}\right)}^{-1}P\left(K=k\right)$$ (10)

whenever $\sum_{i=1}^{n}x_{i}=k$. In a maximum entropy formalism, this model can be obtained by setting *h*_*i*_ = 0 and *J*_*ij*_ = 0 for all *i*, *j* ∈ {1, …, *n*} and only optimising entries of *V*. Without loss of generality, we fixed fixed *V*_0_ = 0 [43], resulting in *n* degrees of freedom for the model.

In flat models, it is possible to explicitly construct a limit *n* → ∞ which will help us understand population analyses performed on experimental data: We assume that there is a spike-count density *f*(*r*), *r* ∈ [0, 1], which describes the population spike-count distribution of an infinitely large population. *f*(*r*) denotes the probability density of a fraction of *r* neurons spiking simultaneously. Finite-size populations of *n* cells are then obtained as random subsamples out of this infinitely large system. Based on previous findings by [25], we show in section 2.3 in S1 Supporting Information that, in this construction, flat models always exhibit a linear divergence of specific heat, unless the limit *f*(*r*) is given by either a single delta peak or a mixture of two symmetric delta peaks. These two models corresponds to systems that (for large *n*) either behave like a fully independent population (whose spike-count distribution converges to a single delta peak), or a population described by a pure pairwise maximum entropy model (which converges to two delta peaks). In particular, any flat model with higher-order correlations [17, 70, 71], or a non-degenerate *f*(*r*), will exhibit ‘signatures of criticality’. Furthermore, we show that, for continuous *f*(*r*), *c*(*T*) does not diverge for any *T* ≠ 1. In combination, these results show that the peak of the specific heat is mathematically bound to converge to *T* = 1 for *n* → ∞ in this model class.

We further simplified the flat model by re-parametrising *P*(*K* = *k*) by a beta-binomial distribution, thereby reducing the number of parameters from *n* to two, and—importantly—obtaining parameters which do not explicitly depend on *n*. In this model,

$$P\left(K=k\right)=\left({}_{k}^{n}\right)\frac{\text{Beta}\left(\alpha +k,\beta +n-k\right)}{\text{Beta}\left(\alpha ,\beta \right)}=\left({}_{k}^{n}\right)\int f\left(r\right)r^{k}{\left(1-r\right)}^{n-k}dr$$ (11)

and

$$\begin{array}{c} f\left(r\right)=\frac{1}{\text{Beta}(\alpha ,\beta )}r^{\alpha -1}{\left(1-r\right)}^{\beta -1}. \end{array}$$ (12)

For simulated data, we found values for *α*, *β* extracted from the beta-binomial fits to populations of different sizes *n* to be stable over a large range of *n* (Fig 3b). We used the beta-binomial parameters obtained from the largest investigated *n* to estimate the divergence rate $\tilde{c}$ for *n* → ∞.

## Supporting information

S1 Supporting Information Supporting derivations and analyses. We provide more detailed descriptions of the maximum entropy fitting procedures used in this study. We derive limiting behavior of specific heat capacity for flat models, and analyze effects of uniform subsampling on sample means and variances. Furthermore, we provide control analyses for central findings of the study.
(PDF)
