# Correction: Signatures of criticality arise from random subsampling in simple population models

**DOI:** 10.1371/journal.pcbi.1005886

**Published:** 2017-12-06

**Authors:** The PLOS Computational Biology Staff

The funding statement for this article should read as follows:

“Work was funded by the German Federal Ministry of Education and Research (BMBF; FKZ: 01GQ1002, Bernstein Center Tübingen, FKZ 01GQ1601 to PB), the German Research Foundation (BE 5601/1-1 to PB; SFB 1233, Robust Vision: Inference Principles and Neural Mechanisms, TP 14 to JHM), the Max Planck Society and the caesar foundation. The funders had no role in study design, data collection and analysis, decision to publish, or preparation of the manuscript.”
