# Supplementary material for: Signatures of criticality arise from random subsampling in simple population models
Source: PLoS Comput Biol. 2017 Oct 3;13(10):e1005718. doi: 10.1371/journal.pcbi.1005718 (PMC5640238; doi:10.1371/journal.pcbi.1005718)
Supplement: S1 Supporting Information — We provide more detailed descriptions of the maximum entropy fitting procedures used in this study. We derive limiting behavior of specific heat capacity for flat models, and analyze effects of uniform subsampling on sample means and variances. Furthermore, we provide control analyses for central findings of the study. (PDF) [file pcbi.1005718.s001.pdf]

Supporting Information

1 Fitting the K-pairwise maximum entropy model to data

To identify the values $\hat{\lambda}$ of the model parameters which yield the best fit of the maximum entropy model to data, we maximise the log-likelihood of the model given the data. The general form of the log-likelihood of a maximum entropy model parametrised by vector λ is given by

$$L(\lambda) = \sum_{m=1}^{M} \log P(\mathbf{x}^{(m)}|\lambda) = -M \log Z_\lambda + \sum_{m=1}^{M} \lambda^\top f(\mathbf{x}^{(m)}) \qquad (1)$$

for the spike-data vectors $x^{(m)} \in \{0,1\}^n$, $m = 1, \ldots, M$ and (usually intractable) normalizer Z_λ,

$$Z_\lambda = \sum_{\mathbf{x}} \exp\left(\lambda^\top f(\mathbf{x})\right).$$

Every choice of the feature function f defines a specific maximum entropy model over this n-dimensional binary space. For the K-pairwise maximum entropy model used in this paper, $f(\mathbf{x}) \in \{0,1\}^{n(n+3)/2+1}$ is composed of:

1. n first-order features

   $$f_i(\mathbf{x}) = x_i,$$

   with corresponding parameters collected in h. The h_i, $i = 1, \ldots, n$ control single-cell firing rates (in units of bins rather than Hz).

2. $n(n-1)/2$ second-order features

   $$f_{ij}(\mathbf{x}) = x_i x_j,$$

   with parameters J_{ij}, $j, i = 1, \ldots, n$, $i < j$, controlling pairwise neuronal correlations.

3. n+1 population-scale features

   $$f_k(\mathbf{x}) = \begin{cases} 1, & \text{if } \sum_i x_i = k \\ 0, & \text{otherwise} \end{cases}$$

   with parameters V_k, $k = 0, \ldots, n$. The vector V controls the overall number of spikes in each temporal bin.

Note that that there is some degeneracy between the parameter vectors V and both h and J —a global upwards shift of firing rates for example can be achieved both by adding a positive constant ϵ to each h_i, or by adding ϵk to each of the V_k. Similarly, adding a constant ϵ to every J_{ij} can be balanced by subtracting $\epsilon\frac{k(k-1)}{2}$ from each V_k. Since either manipulation of V is zero for $k = 0$, fixing $V_{k=0} = 0$ is not sufficient for getting rid of this parameter degeneracy. As we never interpreted the parameter-values

themselves, but only the fit to data, we made no attempt to add additional constraints to achieve a unique parameterization.

We can re-write the K-pairwise model into the general maximum entropy form by stacking the feature functions f_i, f_{ij}, and f_k into the vector-valued feature function f and doing the same with parameters h_i, J_{ij}, and V_k to obtain $\lambda = \{h, J, V\} \in \mathbb{R}^{n(n+3)/2+1}$. The derivative of the log-likelihood with respect to any single parameter $\lambda_l, l = 1, \ldots, n(n+3)/2+1$ is given by (see e.g. [1])

$$\begin{aligned}
\frac{\delta}{\delta\lambda_l}\sum_{m=1}^{M}\log P(\mathbf{x}^{(m)}|\lambda) &= \frac{\delta}{\delta\lambda_l}\sum_{m=1}^{M}\left(\lambda^\top f(\mathbf{x}^{(m)}) - \log Z_\lambda\right) \\
&= \sum_{m=1}^{M}\frac{\delta}{\delta\lambda_l}\lambda^\top f(\mathbf{x}^{(m)}) - \frac{\delta}{\delta\lambda_l}M\log\sum_{\mathbf{x}}\exp\left(\lambda^\top f(\mathbf{x})\right) \\
&= \sum_{m=1}^{M} f_l(\mathbf{x}^{(m)}) - M\frac{\sum_{\mathbf{x}}\lambda_l\exp\left(\lambda^\top f(\mathbf{x})\right)}{\sum_{\mathbf{x}}\exp\left(\lambda^\top F(\mathbf{x})\right)} \\
&= M\left(\frac{1}{M}\sum_{m=1}^{M} f_l(\mathbf{x}^{(m)}) - \mathrm{E}_\lambda[f_l(\mathbf{x})]\right). \qquad (2)
\end{aligned}$$

As can be seen from equation (2), the gradient of the log-likelihood vanishes if and only if the data means match the expectations of $f(\mathbf{x})$ under the model.

To deal with data-sets of limited size, we maximised a regularised variant of the log-likelihood,

$$\begin{aligned}
L(h, J, V|\sigma_h, \sigma_J, \Sigma) :&= \sum_{m=1}^{M}\log P(\mathbf{x}^{(m)}|h, J, V) - \frac{1}{\sigma_h}\|h\|_1 - \frac{1}{\sigma_J}\|J\|_1 - \frac{1}{2}V^\top\Sigma^{-1}V \qquad (3) \\
\Sigma &= (\sigma_S S + \sigma_{\mathbb{I}}\mathbb{I}) - \frac{1}{\sigma_S + \sigma_{\mathbb{I}}}S_{0\bullet}S_{0\bullet}{}^\top \\
S_{kk'} &= \exp\left(-\frac{(k-k')^2}{2\tau_S^2}\right) \\
S_{0k} &= \sigma_S\exp\left(-\frac{k^2}{2\tau_S^2}\right).
\end{aligned}$$

Here, the matrix Σ implements a combined ridge and smoothing regression over V, with $(n+1)\times(n+1)$ identity matrix $\mathbb{I}$ and smoothing matrix S corresponding to a squared-exponential kernel [2]. We set $V_0 = 0$ and accounted for this by conditioning on V_0 and correspondingly subtracted $S_{0\bullet}(\sigma_S + \sigma_{\mathbb{I}})^{-1}S_{0\bullet}{}^\top$ from Σ. We used $\sigma_h = \sigma_J = 10^4$, $\sigma_S = 10$, $\sigma_{\mathbb{I}} = 400$ and $\tau_S = 10$.

To fit maximum entropy models to large neural populations, one needs to first approximate the feature moments $\mathrm{E}_\lambda[f(\mathbf{x})]$ needed for the gradients of both eq. (1) and eq. (3) also for large populations ($n > 20$), and then update the parameters λ.

We introduce two modifications over previous approaches to fitting maximum entropy models to neural data [3] to improve computational efficiency: First, we used pairwise Gibbs sampling and Rao-Blackwellisation to considerably improve estimation of the second-order feature moments $\mathrm{E}_\lambda[f_{ij}(\mathbf{x})]$, and Second, we follow the authors of [4], who described a trick for efficiently updating the parameters in pairwise binary maximum entropy models: If one restricted updates to coordinate-wise updates, then one can calculate the gain from updating a single variable in closed form, which makes it easy to select both the variable to update as well as the step-length in closed form. We show how this trick can be extended to allow a joint update of all the population-count features

V. In addition, the gain in log-likelihood is linear in the feature-moments, which makes it possible to compute it from a running average over the MCMC sample, and avoids having to store the entire sample in memory at any point. We describe our contributions in the sections 1.1 and 1.2, respectively.

1.1 Pairwise Gibbs sampling and Rao-Blackwellisation

Following previous work [1], we used MCMC sampling to approximate the expectations of the feature functions $f(\mathbf{x})$ under the K-pairwise model with parameters λ. These expected values $\mathrm{E}_\lambda[f(\mathbf{x})]$ are required to evaluate the gradients of the (penalised) log-likelihood, as well as the log-likelihood gains resulting from parameter updates. As the number of pairwise terms grows quadratically with population size n, most of the parameters of the model $P(\mathbf{x}|\lambda)$ for large n control pairwise moments $\mathrm{E}_\lambda[x_i x_j]$. To make the estimation of these pairwise interactions more efficient, we implemented a pairwise Gibbs sampler that for each update step of the Markov chain samples two variables x_i and x_j, $i \neq j$, $i, j \in 1, \ldots, n$. This furthermore allowed us to 'Rao-Blackwellise' the single-cell and pair-wise feature components $f_i(\mathbf{x}) = x_i$ and $f_{ij}(\mathbf{x}) = x_i x_j$ [5–8]. i.e. to use the conditional probabilities $P(x_i = 1|x_{\sim i}, \lambda)$ and $P(x_i x_j = 1|x_{\sim\{i,j\}}, \lambda)$ for moment estimation, instead of the binary x_i and $x_i x_j$.

The Rao-Blackwell theorem states that the variance of the Rao-Blackwellized estimators is equal or (as in our case) smaller than that of the original estimators. We construct Rao-Blackwellized estimators from the sampling-based estimators

$$\mathrm{E}_\lambda[f_i(\mathbf{x})] \approx \frac{1}{\tilde{m}} \sum_{m=1}^{\tilde{m}} x_i^{(m)}$$

$$\mathrm{E}_\lambda[f_{ij}(\mathbf{x})] \approx \frac{1}{\tilde{m}} \sum_{m=1}^{\tilde{m}} x_i^{(m)} x_j^{(m)}$$

by conditioning on the transition probabilities $\{P(x_{i^{(m)}}^{(m)}, x_{j^{(m)}}^{(m)} = 1|\mathbf{x}_{\sim\{i^{(m)},j^{(m)}\}}^{(m)}, \lambda)\}_{m=1}^{\tilde{m}}$ used to generate the Markov chain $\{\mathbf{x}^{(m)}\}_{m=1}^{\tilde{m}}$.

Empirically, Rao-Blackwellization resulted in substantially faster convergence of the MCMC-estimated model firing rates $\mathrm{E}_\lambda[f_i(\mathbf{x})]$, second moments $\mathrm{E}_\lambda[f_{ij}(\mathbf{x})]$, and thus also of the covariances $\mathrm{cov}_\lambda(\mathbf{x}_i, \mathbf{x}_j|\lambda) = \mathrm{E}_\lambda[f_{ij}(\mathbf{x})] - \mathrm{E}_\lambda[f_i(\mathbf{x})]\mathrm{E}_\lambda[f_j(\mathbf{x})]$ (see supplementary figure A). Unlike the binary variables x_i, $x_i x_j$ however, the conditional probabilities are real numbers from the interval $(0, 1)$ and cannot be stored in memory-efficient sparse matrices. We thus implemented a running average over conditional probabilities that discards the current chain element immediately after drawing the next one, while keeping track of the quantities

$$\mathrm{E}_\lambda[f_i(\mathbf{x})] \approx \frac{1}{\tilde{m}} \sum_{m=1}^{\tilde{m}} P(x_i^{(m)} = 1 \mid x_{\sim i}^{(m)}, \lambda)$$

$$\mathrm{E}_\lambda[f_{ij}(\mathbf{x})] \approx \frac{1}{\tilde{m}} \sum_{m=1}^{\tilde{m}} P(x_i^{(m)} x_j^{(m)} = 1 \mid x_{\sim\{i,j\}}^{(m)}, \lambda)$$

as $\tilde{m}$ increases from 1 to MCMC sample size $\tilde{M}$. We also kept track of the non-Rao-Blackwellised estimates

$$\mathrm{E}_\lambda[f_k(\mathbf{x})] \approx \frac{1}{\tilde{m}} \sum_{m=1}^{\tilde{m}} \delta\left(\sum_{i=1}^{n} x_i^{(m)}, k\right)$$

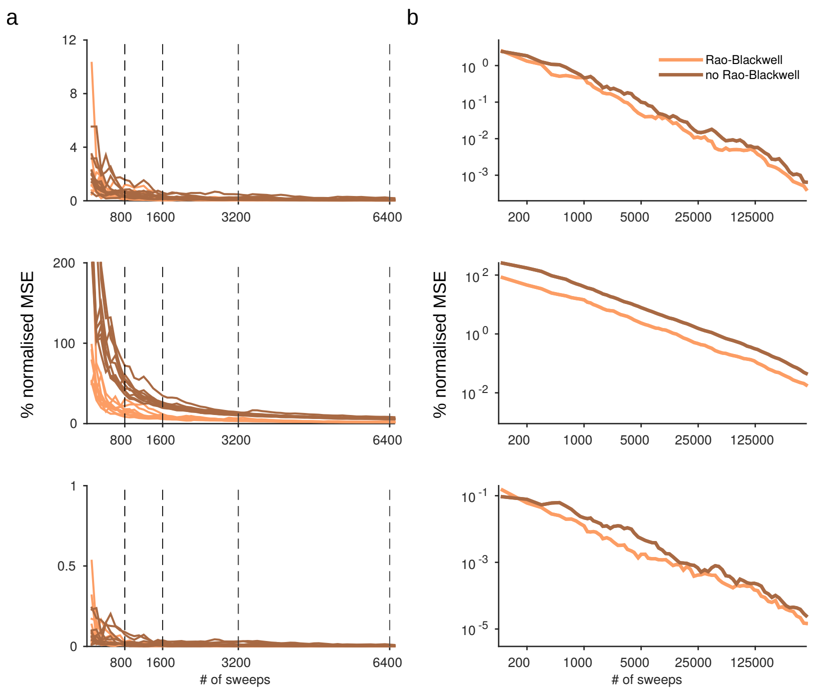

Figure A. Impact of Rao-Blackwellisation a) Comparison of normalised MSE between Rao-Blackwellised and non-Rao-Blackwellised Gibbs sampling, as a function of MCMC chain length, on the 10 subpopulations of size $n = 100$ used in the paper. Top: means, i.e. first-order moments $\mathrm{E}_\lambda[\mathbf{x}_i]$, Center: covariances $\mathrm{cov}_\lambda(\mathbf{x}_i, \mathbf{x}_j)$, Bottom: population-spike count features. No Rao-Blackwellization was used for population-spike count features $P(K = k|\lambda)$. Vertical lines and horizontal axis ticks mark Markov chain lengths used for computing the 1st, 1001st 2001st, ... updates of parameter entries λ_l during training the K-pairwise models to data. All MSEs in this figure are computed as errors between estimated firing rates / covariances / $P(K)$ at given chain length versus the average of the estimates obtained after 10^6 sweeps. **b)** behaviour of MSEs for large MCMC chain lengths. Traces are averages over the 10 traces from panel a.

for the expectations of the population-level indicator feature functions $E_\lambda[f_k(\mathbf{x})] = P(K = k|\lambda)$, with Kronecker delta function $\delta(x, y) = 1$ if $x = y$, and $\delta(x, y) = 0$ otherwise.

We quantified the advantage of Rao-Blackwellising the Gibbs sampler with long Markov chains drawn from the K-pairwise maximum entropy model fits to populations of size $n = 100$ drawn from the simulated RGC data. For each investigated parameter fit, we ran two chains under different conditions: a first chain for which we Rao-Blackwellised the single-cell and pairwise feature moments, and a second chain for which we did not. These Markov chains were run for $\tilde{M} = 10^6$ sweeps and hence orders of magnitude longer than had occurred for the invidivual parameter updates within this study, which comprised 800 to 30000 sweeps, or 3.96×10^6 to 1.485×10^6 individual MCMC chain updates at $n = 100$. The long sample runs served to give an approximation for the "true" expected values of the target quantities of interest to us: firing rates $\mathrm{E}_\lambda[f_i(\mathbf{x})]$, covariances $\mathrm{cov}_\lambda(\mathbf{x}_i, \mathbf{x}_j)$ and population spike count distribution $P(K = k|\lambda)$.

We quantified the speed of convergence of the estimates to the "true" expected feature moments by the normalised MSE between sampler-derived feature moments after any given length $0 < \tilde{m} < \tilde{M}$ of the MCMC chain and the results we got after the full chain

length. After the full $\tilde{M} = 10^6$ sweeps, the Rao-Blackwellised and non-Rao-Blackwellised estimates on average differed by $1.7 \times 10^{-4}\%$, 0.013% and $4 \times 10^{-6}\%$ normalised MSE for firing rates, covariances and population spike count distributions, respectively. We computed the distance to "truth" for each condition as the normalised MSE to the $\mathrm{E}_\lambda[f(\mathbf{x})]$ averaged over both conditions. We obtained MCMC estimates for the feature moments of the K-pairwise maximum entropy models fits to 10 subsampled populations of $n = 100$ neurons each drawn from our retina simulation. Supplementary figure Aa displays the results for the two conditions, Rao-Blackwellised vs. non-Rao-Blackwellised, for each of the 10 investigated fits.

MSEs of firing rate features $\mathrm{E}_\lambda[\mathbf{x}_i]$ did not benefit from Rao-Blackwellisation. This is expected, as each x_i is sampled $n-1$ times per sweep and thus the moments are already well estimated relative to the second-order features. For covariances $\mathrm{cov}_\lambda(\mathbf{x}_i, \mathbf{x}_j)$, normalised MSEs showed clear improvement under Rao-Blackwellisation, visible as an approximately constant offset between the avarages over all 10 parameter fits in the loglog-domain as seen in figure Ab. The normalised MSE on average was 3.19 times higher for non-Rao-Blackwellised (given by the downwards offset of the normalised MSEs of the Rao-Blackwellised estimates). The fraction of samples needed from Rao-Blackwellised runs to achieve the same normalised MSE on the pariwise moments than non-Rao-Blackwellised runs (given by the leftward offset of the normalised MSEs of the Rao-Blackwellised) overall was 32.02%. The fraction ranged from 34.93% at 800 sweeps to 31.74% at 30000 sweeps. The ratio of normalised MSEs was similarly stable, being 2.96 times higher at 800 sweeps and 3.27 times higher at 30000 sweeps for non-Rao-Blackwellised samples than for Rao-Blackwellised ones.

1.2 Exploiting the structure of the K-pairwise feature functions allows blockwise parameter updates.

As described in the previous section, we can use MCMC to obtain the expected values of the feature function $\mathrm{E}_\lambda[f(\mathbf{x})]$ that are needed to to optimise the model parameters λ. To find the parameter setting $\hat{\lambda}$ which maximise the log-likelihood over the given data vectors $\mathbf{x}^{(m)}$, $m = 1, \ldots, M$, we follow an iterative update scheme introduced previously [4], and extend it to the K-pairwise model. The update scheme optimises parameter changes $\lambda^{new} - \lambda^{old}$ relative to a current parameter estimate λ^{old}, rather than the parameters λ directly. The benefit of this scheme over standard gradient ascent on the regularised log-ligkelihood as in eq. (2) is that we can give closed-form solutions for optimal values of a single component λ_l when temporarily holding all other components $\lambda_{\sim l}$ fixed.

Changing the current parameter estimate λ^{old} to λ^{new} leads to a change in log-likelihood of

$$\begin{aligned}\Delta L(\lambda^{new}, \lambda^{old}) &= \frac{1}{M}\sum_{m=1}^{M} \log P(\mathbf{x}^{(m)}|\lambda^{new}) - \frac{1}{M}\sum_{m=1}^{M} \log P(\mathbf{x}^{(m)}|\lambda^{old}) \\ &= (\lambda^{new} - \lambda^{old})^\top \left(\frac{1}{M}\sum_{m=1}^{M} f(\mathbf{x}^{(m)})\right) - \mathrm{E}_{\lambda^{old}}\left[\exp\left((\lambda^{new} - \lambda^{old})^\top f(\mathbf{x})\right)\right].\end{aligned} \quad (4)$$

The only relevant expectations are w.r.t. the data distribution and $P(\mathbf{x}|\lambda^{old})$, i.e. the current parameter estimate. The term $\mathrm{E}_{\lambda^{old}}[\exp\left((\lambda^{new} - \lambda^{old})^\top f(\mathbf{x})\right)]$ can be simplified when restricting the update vector $\lambda^{new} - \lambda^{old}$ to be non-zero only in selected components. In the simplest case, only a single component λ_l is updated. In this case, the fact that all components of the K-pairwise feature function $f(\mathbf{x})$ are binary, allows to move the

exponent out of the expected value, a trick used by [4]:
The resulting single-coordinate updates only require the feature moments $\mathrm{E}_{\lambda^{old}}[f_l(\mathbf{x})]$:

$$\begin{aligned}\mathrm{E}_{\lambda^{old}}[\exp\left((\lambda^{new}-\lambda^{old})^\top f(\mathbf{x})\right)] &= \mathrm{E}_{\lambda^{old}}[\exp((\lambda_l^{new}-\lambda_l^{old})f_l(\mathbf{x})] \\ &= \mathrm{E}_{\lambda_{old}}[1+(\exp(\lambda_l^{new}-\lambda_l^{old})-1)f_l(\mathbf{x})] \\ &= 1+(\exp(\lambda_l^{new}-\lambda_l^{old})-1)\mathrm{E}_{\lambda_{old}}[f_l(\mathbf{x})].\end{aligned}$$

Equation (4) can now be solved analytically for the single free component λ_l^{new} that maximises the change in log-likelihood. A closed-form optimal solution is still possible when adding an $l1$-penalty to the log-likelihood [4]. We use this $l1$-regularised variant to calculate the possible gain in penalized log-likelihood for each possible update of the single-cell (h_i) and pairwise (J_{ij}) feature moments $\mathrm{E}[x_i]$ and $\mathrm{E}[x_i x_j]$, and then perform the update which yield the largest gain.

If we instead allow more than a single component l of the update $\lambda_l^{new}-\lambda_l^{old}$ to be non-zero, we in general would have to deal with the term

$$\mathrm{E}_{\lambda_{old}}\left[\prod_{l\in J}[1+(\exp(\lambda_l^{new}-\lambda_l^{old})-1)f_l(\mathbf{x})]\right],$$

which requires the higher-order moments $\mathrm{E}_{\lambda_{old}}\left[\prod_{l\in I}f_l(\mathbf{x})\right]$ for all $I\subseteq J$ and $J\subseteq\{1,\ldots,n\}$ being the index set of components that are not set to zero.

The population spike-count features $f_k(\mathbf{x})$, however, are mutually exclusive (only one of the n+1 features can be non-zero at any time), and therefore we can update all parameters of V jointly, and still pull the exponenetial term outside of the expectation. For the population-spike count features $f_k(\mathbf{x})$, hereafter collectively called $f^V(x)\in\{0,1\}^{n+1}$, all such terms of order $||I||>1$ are zero due to the sparsity of $f^V(x)$. When restricting the current parameter update of λ to only update components corresponding to V, we have

$$\Delta L(V^{new},V^{old})=(V^{new}-V^{old})^\top\left(\frac{1}{M}\sum_{m=1}^{m}f^V(\mathbf{x}^{(m)})\right)-\mathrm{E}_{\lambda^{old}}[\exp((V^{new}-V^{old})^\top f^V(\mathbf{x}))]$$

and

$$\begin{aligned}\mathrm{E}_{\lambda^{old}}[\exp\left((V^{new}-V^{old})^\top f^K(\mathbf{x})\right)] &= \sum_{\mathbf{x}}\exp\left((V^{new}-V^{old})^\top f^K(\mathbf{x})\right)P(\mathbf{x}|\lambda^{old}) \\ &= \sum_{k=0}^{n}\sum_{\mathbf{x}:\sum_i x_i=k}\exp\left((V_k^{new}-V_k^{old}f_k^K(\mathbf{x})\right)P(\mathbf{x}|\lambda^{old}) \\ &= \sum_{k=0}^{n}\exp\left((V_k^{new}-V_k^{old})f_k^K(\mathbf{x})\right)\sum_{\mathbf{x}:\sum_i x_i=k}P(\mathbf{x}|\lambda^{old}) \\ &= \sum_{k=0}^{n}\exp\left((V_k^{new}-V_k^{old})f_k^K(\mathbf{x})\right)P(k|\lambda^{old}).\end{aligned}$$

We obtained estimates of the values of $P(k|\lambda^{old})=\mathrm{E}_{\lambda^{old}}[f_k(\mathbf{x})]$ from the MCMC sample using the indicator functions $f_k(\mathbf{x})$, and optimising w.r.t. V_k^{new}, $k\in\{1,\ldots,n\}$ using gradient-based methods [9].

In summary, our update-scheme for maximising the log-likelihood proceeds as follows: For a given parameter vector λ^{old}, we first estimate the expectation of the feature functions $f_i(\mathbf{x})$, $f_{ij}(\mathbf{x})$ and $f_k(\mathbf{x})$ using a running average over an MCMC sampling and Rao-Blackwellization. We then calculate, for each possible single-neuron parameter h_i

and each possibly pairwise term J_{ij} the gain in penalised log-likelihood that we would get from updating it, using methods as described above and derived in [4]. We additionally compute the gain in penalised log-likelihood that would result from optimising all n of the free V parameters jointly, using a convex optimization. Finally, we choose the update that brings the largest gain, and either update a single h_i, a single J_{ij}, or all V parameters. Subsequently, we again estimate the new feature functions using MCMC sampling given the current estimate of $\lambda^{old} \leftarrow \lambda^{new}$ before we update again. We initialised the algorithm assuming independent neurons (i.e. setting each h_i using the firing rate of each neuron, and leaving J and V zero). The algorithm then typically first updated all V parameters, before proceeding to jump between different J, h and V updates.

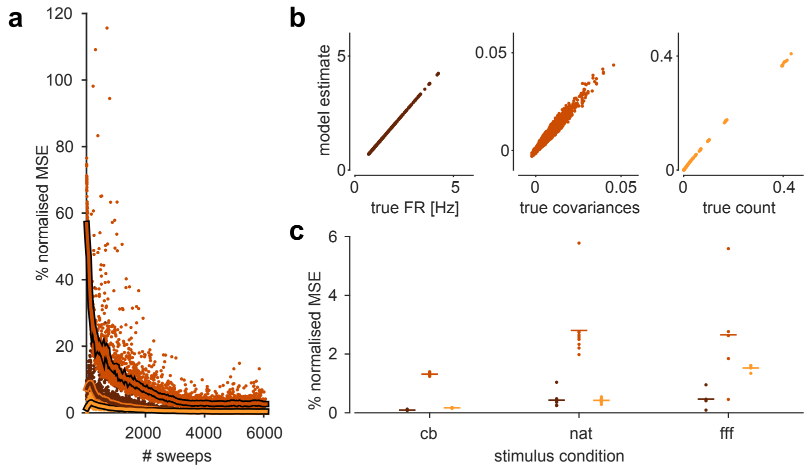

Figure B. Quality of fits for K-pairwise maximum entropy model across multiple populations and stimulus conditions a) Normalised MSEs for firing rates, covariances and $P(K)$ during parameter learning. Error values collapses across 10 subpopulations at $n = 100$, fit to simulated activity in response to natural images, one point for each displayed iteration and each subpopulation. Lines are moving averages (smoothing kernel width = 150 param. updates). **b)** Quality of fit after parameter learning. Data vs. model estimates for firing rates, covariances and $P(K)$, collapsed over all 10 subpoplations with size $n = 100$. **c)** Quality of fit for different stimulus types. Normalised MSEs after maximum entropy model fitting shown for 10 subpopulations for natural images (nat) and 5 subpopulations each for checkerboard (cb) and full-field flicker (fff). All subpopulations of size $n = 100$. Vertical bars give averages. Colours as in **a), b)**.

1.3 Effect of temperature on K-pairwise model statistics

We compute specific heat curves from the K-pairwise model by introducing a temperature T that scales the learned parameters by $\frac{1}{T}$, i.e. $\lambda_T = \lambda/T$. Temperature $T = 1$ corresponds to the statistics of the empirical data. By changing T to other parameter values one can perturb the statistics of the system [10]: For models fit to our simulated data, increasing temperature leads to models with higher firing rates and weaker correlations (Fig. C), with $P_T(\mathbf{x})$ approaching the uniform distribution for very large T. If the temperature is decreased towards zero, $P_T(\mathbf{x})$ has most of its probability mass over the most probable spike patterns. In many probabilistic systems, lowering T leads to increasing correlations, as the systems then 'jumps' between several different patterns

and thus the activation probabilities of different elements are strongly dependent on each other. However, for the simulated RGC activity, the qualitative behaviour is different, and the sparsity of data leads to a decrease of correlations: At a bin size of 20 ms [11], the most probable state is the silent state, followed by patterns in which exactly one neurons spikes. In an example population of size $n = 100$, 53.8% of observed spike patterns contain at most one spike. When decreasing the temperature to $T < 1$, patterns with at most one spike dominate the systems even more strongly: For the same population and temperature $T = 0.8$, we find 95.6% of observed patterns to contain at most one spike. Thus, when the temperature is lowered, the shift in probability mass to single-spike patterns decreases correlations.

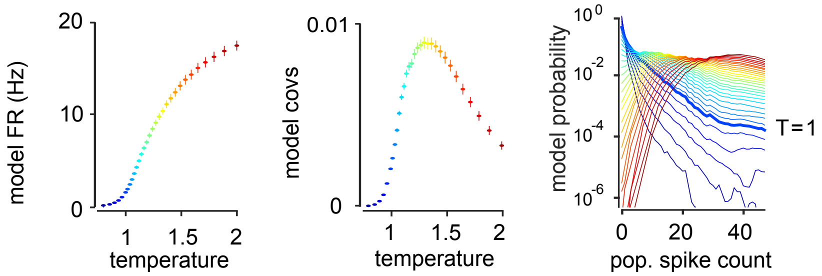

Figure C. Temperature and population statistics for K-pairwise models Changing the temperature parameter scales firing rates (left), covariances (centre) and population spike-counts (right) of samples $\mathbf{x}$ generated from temperature-perturbed K-pairwise model fits.

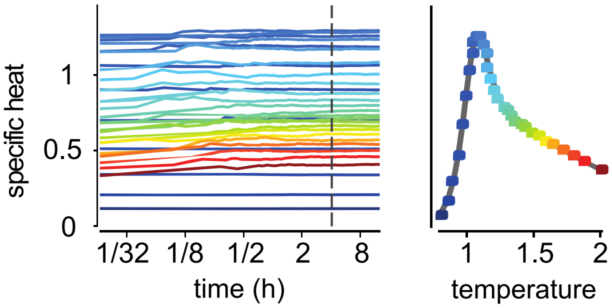

Figure D. Estimating specific heat via MCMC sampling MCMC estimates of specific heat from a K-pairwise maximum entropy model fit to example population with $n = 100$. For every investigated temperature $0.8 \leq T \leq 2$, we run a Markov Chain to sample from the temperature-perturbed maximum entropy model. Estimates were taken from the average over first 4h computation time of sampling (dashed vertical line).

2 Supplementary Text: Specific heat in simple models

We refer to a maxmimum entropy model as 'flat' if it is fully specified by the population spike count distribution $P(\sum_{i=1}^{n} x_i = k)$, i.e. the model class studied in [12–14]. In this model class, all neurons have the same firing rate μ and pairwise correlation ρ. As neuron identities become interchangeable, all $\binom{n}{k}$ possible patterns $\mathbf{x}$ with $\sum_{i=1}^{n} = k$ are

assigned the same probability $P(k) = P(\mathbf{x})\binom{n}{k}$. In flat models, all relevant population properties can be computed from summing over $n+1$ different spike counts, and one never has to (explicitly) sum over the entire 2^n possible spike patterns.

2.1 Independent neurons

A special case of a flat model is an independent model in which all neurons have the same firing rates and zero correlations. Assuming independent spiking for each of the n neurons and a shared probability $q \in [0,1]$ to fire in a time bin, the distribution of population spike counts $k = \sum_{i=1}^{n} x_i$ is given by a binomial distribution,

$$P(\mathbf{x}|q) = q^k (1-q)^{n-k}$$
$$P(k|q) = \binom{n}{k} q^k (1-q)^{n-k}.$$

To compute specific heat capacities for the underlying neural population of size n, we can rewrite the binomial distribution in maximum entropy form

$$P(\mathbf{x}|V) = \frac{1}{Z(V)} \exp(V_k)$$
$$P(k|V) = \frac{1}{Z(V)} \binom{n}{k} \exp(V_k).$$

Re-introducing parameters V_k, $k = 0, \ldots, n$, we find

$$\begin{aligned} V_k &= \log P(k|q) - \log \binom{n}{k} + \log Z(V) \\ &= k \log(q) + (n-k) \log(1-q)), \end{aligned}$$

and for the heat capacity, we get

$$\begin{aligned} \mathrm{Var}[\log P(x|V)] &= \mathrm{Var}[k \log(q) + (n-k) \log(1-q)] \\ &= (\log(q) - \log(1-q))^2 \ \mathrm{Var}[k]. \end{aligned}$$

The binomial variance is $\mathrm{Var}[k] = nq(1-q)$. We plug this in and see that at unit temperature $T = 1$, the specific heat is given by

$$c(T=1) = \frac{1}{n} \mathrm{Var}[\log P(\mathbf{x}|V)] = q(1-q)(\log(q) - \log(1-q))^2, \tag{5}$$

which is independent of population size n.

When explicitly introducing temperatures other than $T = 1$, we add a factor $\frac{1}{T} = \beta$ that scales the parameters V and renormalise, yielding

$$P(k|V,T) = \frac{1}{Z(\beta V)} \binom{n}{k} \exp(\beta V_k),$$

where V_k, $k = 0$, ..., n is defined w.r.t. q as above. This is the same functional form as was given for the binomial distribution at $T = 1$, with only parameters V being replaced by βV. We can also go back to the standard binomial parametrisation with $q_\beta = \frac{q^\beta}{q^\beta + (1-q)^\beta}$ and obtain

$$P(k|V,T) = \binom{n}{k} q_\beta^k (1-q_\beta)^{(n-k)}.$$

Changing the temperature $T = \frac{1}{\beta}$ retains the binomial form of the population model, and we can generalise the expression for the specific heat (5) of the independent flat model for any temperature T to be

$$c(T) = q_\beta(1 - q_\beta)(\log(q_\beta) - \log(1 - q_\beta))^2, \tag{6}$$

which again is independent of the population size n. The independent flat model is a case that does not show divergent specific heat, and for which the peak of the heat is not necessarily at unit temperature. Later, we will derive why this makes the binomial model one of only two non-critical special cases.

The independent model also allows to identify the location of the peak specific heat, $c(T^*)$, for $q \neq 0.5$. We have

$$\frac{\delta c}{\delta \beta} = \beta q_\beta \left(1 - q_\beta\right) \log^2 \left(\frac{q}{1-q}\right) \left(2 + \beta \log(\frac{q}{1-q}) \left(1 - 2q_\beta\right)\right),$$

which for a given q has a root at β such that

$$\frac{q^\beta + (1-q)^\beta}{q^\beta - (1-q)^\beta} = \frac{1}{2} \log \left(\frac{q^\beta}{(1-q)^\beta}\right).$$

An interesting question to ask is on which side of the unit temperature $T = \beta = 1$ the peak of the specific heat is found. In this case, we seek a q such that the peak specific heat is found at $\beta = 1$. Note that these specific values for q come in pairs q, $1 - q$. In the context of binned spiking activity, we will focus on the smaller value in each such pair. For the peak to be at unit temperature $\beta = 1$, we require

$$q = (1-q) \exp \left(\frac{1}{q - \frac{1}{2}}\right) \approx 0.0832. \tag{7}$$

Assuming 20 ms temporal binning of activity data, this corresponds to an average firing rate of 4.16 Hz – higher than any firing rate in our simulated RGC population. The peak specific heat for independent populations (irregardless of the population size) with a firing rate below this value are found for $T^* > 1$ (see Fig. 3f). Populations with higher average firing rate have the peak specific heat at a temperature $T^* < 1$.

For the more general case of independent neurons but with different firing rates, we have

$$c(\beta) = \frac{1}{n} \sum_{i=1}^{n} q_\beta^{(i)}(1 - q_\beta^{(i)})(\log(q_\beta^{(i)}) - \log(1 - q_\beta^{(i)}))^2,$$

for $q^{(i)} = \frac{\exp(h_i)}{1+\exp(h_i)}$ and $q_\beta^{(i)} = \left(q^{(i)}\right)^\beta / (\left(q^{(i)}\right)^\beta + \left(1 - q^{(i)}\right)^\beta)$.

2.2 Aside: Asymptotic entropy in flat models

To calculate the variance of log-probabilities, we first need the mean log-probability, i.e. the (negative) entropy.

Entropy: Recalling that $P(k) = P(\mathbf{x})\binom{n}{k}$, the entropy of the flat model for general $P(k)$ can be written as

$$\begin{aligned} H_n &= -\sum_x P(\mathbf{x}) \log P(\mathbf{x}) \\ &= -\sum_k \sum_{\mathbf{x}: \sum_i x_i = k} P(\mathbf{x}) \log P(\mathbf{x}) \\ &= -\sum_k P(k) \left(\log P(k) - \log \binom{n}{k} \right). \end{aligned}$$

Thus, the entropy of a flat model is

$$H_n = -\sum_k P(k) \left(\log P(k) - \log \binom{n}{k} \right).$$

Asymptotic entropy: We assume that $P(k)$ has a limiting distribution $f(r)$, where $r \in [0, 1]$ is the probability density of a proportion of r neurons spiking simultaneously. Therefore, for large n

$$\begin{aligned} H_n &= -\sum_k P(k) \left(\log P(k) - \log \binom{n}{k} \right) \\ &\approx -\sum_k \frac{1}{n} f\left(\frac{k}{n}\right) \left(\log P(k) - \log \binom{n}{k} \right) \\ &\approx -\int_0^1 f(r) \left(\log \frac{f(r)}{n} - \log \binom{n}{nr} \right) dr \\ &= -\int_0^1 f(r) \log f(r) dr + \log(n) + n \int_0^1 f(r) \eta(r) dr. \end{aligned}$$

Here, we used Stirling's approximation to obtain, for large n,

$$\log \binom{n}{nr} \approx n \left(-r \log r - (1-r) \log(1-r) \right) =: n\eta(r). \tag{8}$$

As the first term is constant in n, the second term only grows with $\log(n)$, and the third with n, we get that the entropy of a flat model, for large n, is given by

$$H_n = nh, \tag{9}$$

with

$$h := \int_0^1 f(r) \eta(r) dr. \tag{10}$$

2.3 Asymptotic specific heat in flat models at unit temperature

Next, we calculate the specific heat, first exactly and then for large n, and finally for weakly correlated models:

First, the specific heat is given by

$$\begin{aligned} c(T=1) &= \frac{1}{n} \mathrm{Var}[\log P(\mathbf{x})] = \frac{1}{n} \sum_x P(\mathbf{x}) \left(\log P(\mathbf{x}) - \mathrm{E}[\log P(\mathbf{x})] \right)^2 \\ &= \frac{1}{n} \sum_k P(k) \left(\log P(k) - \log \binom{n}{k} - \mathrm{E}[\log P(\mathbf{x})] \right)^2. \end{aligned}$$

Using $\mathrm{E}[\log P(\mathbf{x})] = -H_n$, we get that

$$c(T=1) = \frac{1}{n}\sum_k P(k)\left(\log P(k) - \log\binom{n}{k} + H_n\right)^2 \text{ or}$$
$$= \frac{1}{n}\sum_k P(k)\left(\log P(k) - \log\binom{n}{k}\right) - \frac{1}{n}H_n^2.$$

For large n, we have that $P(k) \approx \frac{1}{n} f\left(\frac{k}{n}\right)$. We get that

$$\begin{aligned}
c(T=1) &= \frac{1}{n}\sum_k P(k)\left(\log P(k) - \log\binom{n}{k} + H_n\right)^2 \\
&\approx \frac{1}{n}\sum_k \frac{1}{n} f\left(\frac{k}{n}\right)\left(\log\left(\frac{1}{n} f\left(\frac{k}{n}\right)\right) - \log\binom{n}{k} + H_n\right)^2 \\
&\approx \frac{1}{n}\int_0^1 f(r)\left(\log f(r) - \log n - \log\binom{n}{nr} + H_n\right)^2 dr \\
&\approx \frac{1}{n}\int_0^1 f(r)\left(\log f(r) - \log n - n\eta(r) + nh_n\right)^2 dr \\
&= \frac{1}{n}\int_0^1 f(r)\left((\log f(r) - \log n)^2 + n^2\left(\eta(r) - h_N\right)^2 + 2n\left(\log f(r) - \log n\right)\left(h_n - \eta(r)\right)\right) dr \\
&= \frac{1}{n}\int_0^1 f(r)\left(\log^2 f(r) + \log f(r)\left(2n\left(h_n - \eta(r)\right) - 2\log n\right)\right) dr \\
&+ \frac{1}{n}\int_0^1 f(r)\left(\log^2 n - n^2\left(h_n - \eta(r)\right)\right) dr.
\end{aligned}$$

For large n, this integral is dominated by the term in n^2, and thus the specific heat is asymptotically given by

$$c(T=1) = n\int_0^1 f(r)\left(\eta(r) - h\right)^2 dr. \tag{11}$$

Therefore, in general, the specific heat grows linearly, and hence diverges (see Fig. G). The only exception to this are models for which $\eta(r) - h_n = 0$ for almost all r. This happens if $f(r)$ is a delta-distribution, $f(r) = \delta(r-\mu)$, in which case $h_n = \eta(\mu)$ and therefore the integral vanishes. This occurs whenever the pairwise correlations do not grow proportionally with n^2, as then the variance of the population spike count collapses in the limit. One such special case is the binomial distribution over k, as already demonstrated above using a more direct approach. There is a second special case, namely if $f(r)$ is a combination of two δ-peaks at μ and $1-\mu$ (See [13] for details)– this special case corresponds to a flat Ising model.

2.4 In flat models, specific heat does not diverge for temperatures which are not equal to 1:

Above we showed that at unit temperature, the specific heat for flat models (almost) always diverges. Now, we show that this is NOT true for any other temperature. This explains that, for any $f(r)$, we will find that the unit temperature is 'special'.

First, we calculate the spike-count distribution at any inverse temperature β:

$$P_\beta(\mathbf{x}) = \frac{1}{Z_\beta} P(\mathbf{x})^\beta$$
$$P_\beta(k) = \frac{1}{Z_\beta} \binom{n}{k}^{1-\beta} P(k)^\beta.$$

For large n,

$$\begin{aligned} f_\beta(r) &\approx n P_\beta(rn) \\ &= \frac{n}{Z_\beta} \binom{n}{nr}^{1-\beta} P(rn)^\beta \\ &\approx \frac{n}{Z_\beta} \exp\left(n(1-\beta)\eta(r)\right) P^\beta(rn). \end{aligned}$$

For large populations, this expression is dominated by the exponential $\exp\left(n(1-\beta)\eta(r)\right)$. For $\beta < 1$, the exponential term is in turn dominated by the mode of $\eta(r)$, which is at $r = \frac{1}{2}$. Thus, for $\beta < 1$, $f_\beta(r) = \delta(r - \frac{1}{2})$, a delta-peak at $r = \frac{1}{2}$.
Conversely, for $\beta > 1$, the argument of the exponential has its peaks at $r = 0$ and $r = 1$, and therefore $f_\beta(r) = \frac{1}{2}\delta(r-1) + \frac{1}{2}\delta(r-0)$. In this case, we also have that the integral in the specific heat vanishes, and that the specific heat does not diverge.

3 Specific heat divergence rate in flat models as function of correlation strength

In the next two sections, we will derive analytic expressions to predict the specific heat divergence rate in flat models as a function of the correlation strength within the population. Starting out from eq. (11), we will use two different approximations to $f(r)$ that will each yield results that allow us to better understand the behaviour of the specific heat at unit temperature $c(T=1)$ in flat models.

3.1 Asymptotic entropy and specific heat in weakly correlated flat models:

Next, we examine entropy and specific heat in models with weak correlations. If the model is weakly correlated and its mode is not at 0 or 1 we can assume it to be approximately Gaussian with mean μ and variance σ^2,

$$f(r) = \frac{1}{Z} \exp\left(-\frac{1}{2\sigma^2}(r-\mu)^2\right).$$

We first calculate the entropy: We expand $\eta(r)$ to second order around μ,

$$\begin{aligned} \eta(r) &= \eta(\mu+\delta) = \eta(\mu) + \eta'(\mu)\delta + \frac{\delta^2}{2}\eta''(\mu) + ..., \text{where} \\ \eta'(r) &= \log\left(\frac{1-r}{r}\right) \\ \eta''(r) &= \frac{-1}{r(1-r)}, \end{aligned}$$

hence

$$\eta(\mu+\delta) = \eta(\mu) + \delta \log\left(\frac{1-\mu}{\mu}\right) - \frac{\delta^2}{2\mu(1-\mu)} + \dots$$
$$=: \alpha + \delta\beta + \delta^2\gamma.$$

Thus, the asymptotic entropy-rate is given by

$$\begin{aligned} h &= \int f(r)\eta(r)dr \\ &= \alpha + 0\beta + \gamma\sigma^2 \\ &= \eta(\mu) - \frac{1}{2\mu(1-\mu)}\sigma^2. \end{aligned}$$

We further investigate the variance, again neglecting all terms which are of higher order than 2, obtaining

$$\begin{aligned} (\eta(\mu+\delta) - h)^2 &= \left((\alpha - h) + \beta\delta + \gamma\delta^2\right)^2 \\ &= (\alpha-h)^2 + \delta^2\beta^2 + 2(\alpha-h)\beta\delta + 2(\alpha-h)\gamma\delta^2 + 2(\alpha-h)\gamma\delta^2 + \dots \\ &= (\alpha-h)^2 + \delta\left(2(\alpha-h)\beta\right) + \delta^2\left(\beta^2 + 2(\alpha-h)\gamma\right) + \dots \end{aligned}$$

Integrating this expression over $f(r)$, and dropping all terms in σ which are of order higher than 2, we get

$$\begin{aligned} \int f(r)(\eta(r)-h)^2 &= (\alpha-h)^2 + \sigma^2\left(\beta^2 + 2(\alpha-h)\gamma\right) \\ &= \frac{\sigma^4}{\mu^2(1-\mu)^2} + \sigma^2\left(\log^2\left(\frac{1-\mu}{\mu}\right) - \frac{\sigma^2}{\mu^2(1-\mu)^2}\right) \\ &\approx \sigma^2\log^2\left(\frac{1-\mu}{\mu}\right). \end{aligned}$$

In summary, we arrive at

$$c(T=1) = n\sigma^2\log^2\left(\frac{1-\mu}{\mu}\right), \tag{12}$$

and for small μ

$$c(T=1) = n\sigma^2\log^2(\mu).$$

In other words, a population of a given size n at fixed firing rate μ that has a high specific heat is simply a population which is very correlated. Inspecting the equations above, we see that the final results do not critically depend on the Gaussian assumption—the only requirement for the calculation to be accurate is that the distribution is reasonably peaked around its mean.

3.2 Asymptotic specific heat in the beta-binomial population model

For the beta-binomial model, we assume $f(r)$ to be given by a beta distribution, i.e.

$$f(r) = \frac{1}{B(\alpha,\beta)} r^{\alpha-1}(1-r)^{\beta-1}.$$

Such $f(r)$ arise for large populations when the population spike count k is described by a beta-binomial distribution, and the choice for the beta distribution as a model for $f(r)$ was motivated by the successful application of beta-binomial models $P(k|\alpha,\beta)$ to our simulated RGC activity (see Fig. E, F).

For beta-distributed r, we have

$$\mathrm{E}[r] = \frac{\alpha}{\alpha+\beta},$$
$$\mathrm{Var}[r] = \frac{\alpha\beta}{(\alpha+\beta)^2(\alpha+\beta+1)},$$
$$\mathrm{E}[\log r] = \gamma(\alpha) - \gamma(\alpha+\beta),$$

where γ denotes the digamma function.

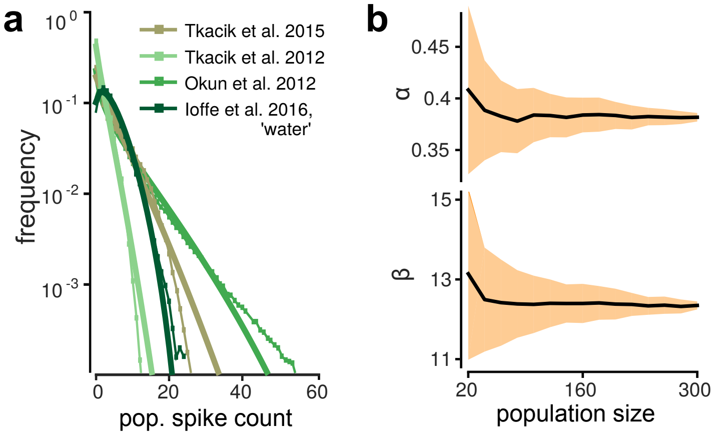

Figure E. Beta-binomial models fit to spike-count distributions from literature and our retina simulation a) Spike-count distributions published in previous studies (dotted) and corresponding beta-binomial fits. As respective population sizes for the beta-binomial distribution, we used the reported $n = 120$ for Tkacik et al. (2015), $n = 40$ for Tkacik et al. (2012), $n = 96$ for Okun et al. (2012), and $n = 128$ for Ioffe and Berry (2016). **b)** Beta-binomial parameters α, β for population subsampled from our RGC simulation with different sizes n. Means $\pm$ 1 standard deviation over 100 random subsamples per population size.

The entropy can be calculated using known results on the expectation of the log,

$$h = \gamma(\alpha+\beta+1) - \frac{\alpha}{\alpha+\beta}\gamma(\alpha+1) - \frac{\beta}{\alpha+\beta}\gamma(\beta+1).$$

For the specific heat at unit temperature according to equation (11), we however also require the expected values

$$\mathrm{E}[r^2\log^2 r], \mathrm{E}[(1-r)^2\log^2(1-r)], \mathrm{E}[r(1-r)\log r\log(1-r)], \tag{13}$$

i.e.

$$\mathrm{E}[r^k(1-r)^l\log^m r\log^n(1-r)] = \int_0^1 f(r)\{r^k(1-r)^l\log^m r\log^n(1-r)\}dr \tag{14}$$

under beta-binomial distribution $f(r)$, where $k, l, m, n \in \{0, 1, 2\}$.

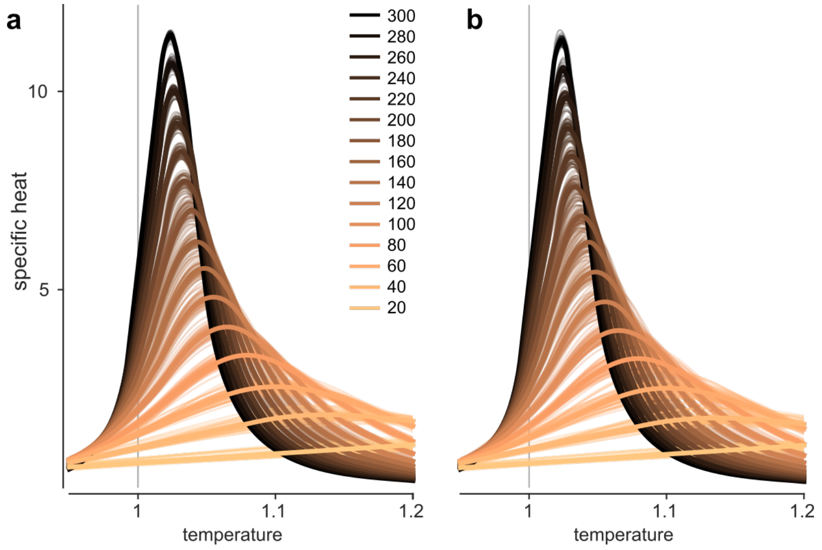

Figure F. (No) Influence of beta-binomial approximation on heat capacity Specific heat capacities computed from population spike count distributions $P(K)$. Spike count distributions for population sizes $n = 20, \ldots, 300$ were obtained from 50 uniformly drawn subpopulations each. Simulated retinal activity was taken from of the retina simulation with in total $N = 316$ RGCs that responded to natural image stimulation. Resulting specific heat traces computed from **a**) beta-binomial approximations to the spike count distributions and **b**) from raw $P(K)$ (right) do not display strong qualitative differences.

We begin the derivation of these terms by observing that

$$\begin{aligned}
u(m,n)(r,\alpha+k,\beta+l) &= \log(r)^m r^{(\alpha+k-1)} \log(1-r)^n (1-r)^{(\beta+l-1)}, \\
\frac{\delta}{\delta\alpha} u_{(m,n)}(r,\alpha+k,\beta+l) &= \log(r)^{m+1} r^{(\alpha+k-1)} \log(1-r)^n (1-r)^{(\beta+l-1)} \\
&= u_{(m+1,n)}(r,\alpha+k,\beta+l), \\
\frac{\delta}{\delta\beta} u_{(m,n)}(r,\alpha+k,\beta+l) &= \log(r)^m r^{(\alpha+k-1)} \log(1-r)^{n+1} (1-r)^{(\beta+l-1)} \\
&= u_{(m,n+1)}(r,\alpha+k,\beta+l),
\end{aligned}$$

for any $k, l \in \mathbb{N}$. Note that the exponents k, l are readily absorbed into new effective beta distribution parameters $\alpha' = \alpha + k$, $\beta' = \beta + l$.

The triplets ($u_{(m,n)}$, $u_{(m+1,n)}$,$u_{(m,n+1)}$) for any $m, n \in \mathbb{N}$ recursively express the integrands of (14) as continuous derivatives, which allows us to repeatedly apply Leibniz' rule to the integral. We first deal with $\mathrm{E}[r^k \log^m r]$, where $m = k = 2$, $n = l = 0$, $\alpha' = \alpha + 2$, $\beta' = \beta$, which is the first of the three expected values we need to compute

the specific heat at unit temperature:

$$\begin{aligned}
\mathrm{Beta}(\alpha,\beta)\mathrm{E}[r^2\log^2 r] &= \int_0^1 r^{\alpha-1}(1-r)^{\beta-1}\log^2(r)r^2dr \\
&= \int_0^1 \frac{\delta^2}{\delta\alpha^2}\{r^{\alpha+1}(1-r)^{\beta-1}\}dr \\
&= \int_0^1 \frac{\delta}{\delta\alpha}\{\frac{\delta}{\delta\alpha}\{r^{\alpha+1}(1-r)^{\beta-1}\}\}dr \\
&= \frac{\delta}{\delta\alpha}\int_0^1 \frac{\delta}{\delta\alpha}\{r^{\alpha+1}(1-r)^{\beta-1}\}dr \\
&= \frac{\delta^2}{\delta\alpha^2}\int_0^1 r^{\alpha+1}(1-r)^{\beta-1}dr \\
&= \frac{\delta^2}{\delta\alpha^2}\mathrm{Beta}(\alpha+2,\beta).
\end{aligned}$$

The first two derivatives of $\mathrm{Beta}(\alpha',\beta')$ w.r.t. α are given by

$$\begin{aligned}
&\frac{\delta}{\delta\alpha}\mathrm{Beta}(\alpha',\beta') = \mathrm{Beta}(\alpha',\beta')(\psi_0(\alpha')-\psi_0(\alpha'+\beta')), \\
&\frac{\delta^2}{\delta\alpha^2}\mathrm{Beta}(\alpha',\beta') = \mathrm{Beta}(\alpha',\beta')\left((\psi_0(\alpha')-\psi_0(\alpha'+\beta'))^2+\psi_1(\alpha')-\psi_1(\alpha'+\beta')\right).
\end{aligned}$$

We obtain the m-th derivative also for $m > 2$ using an iterative rule. The beta-binomial normaliser $\mathrm{Beta}(\alpha',\beta')$ furthermore cancels out with the denominator $\mathrm{Beta}(\alpha,\beta)$ of the original beta distribution through

$$\mathrm{Beta}(\alpha+k,\beta+l) = \frac{\prod_{i=0}^{k-1}(\alpha+i)\prod_{j=0}^{l-1}(\beta+j)}{\prod_{i=0}^{k+l-1}(\alpha+\beta+i)}\mathrm{Beta}(\alpha,\beta).$$

Combining the previous results gives

$$\begin{aligned}
\mathrm{E}[r^2\log^2 r] &= \frac{1}{\mathrm{Beta}(\alpha,\beta)}\int_0^1 r^{\alpha-1}(1-r)^{\beta-1}log^2(r)r^2dr \qquad (15)\\
&= \frac{1}{\mathrm{Beta}(\alpha,\beta)}\frac{\delta^2}{\delta\alpha^2}\mathrm{Beta}(\alpha+2,\beta) \\
&= \frac{\alpha(\alpha+1)}{(\alpha+\beta)(\alpha+\beta+1)}\frac{1}{\mathrm{Beta}(\alpha+2,\beta)}\frac{\delta^2}{\delta\alpha^2}\mathrm{Beta}(\alpha+2,\beta) \\
&= \frac{\alpha(\alpha+1)\left((\psi_0(\alpha+2)-\psi_0(\alpha+\beta+2))^2+\psi_1(\alpha+2)-\psi_1(\alpha+\beta+2)\right)}{(\alpha+\beta)(\alpha+\beta+1)}.
\end{aligned}$$

For $m = 2$, $k = 1$, $n, l = 0$ the result

$$E[r\log^2 r] = \frac{\alpha}{\alpha+\beta}[(\psi_0(\alpha+1)-\psi_0(\alpha+\beta+1))^2+\psi_1(\alpha+1)-\psi_1(\alpha+\beta+1)]$$

is identical to the one from [15] in the appendix A.3, eq. (28).

We have $\mathrm{Beta}(\alpha,\beta) = \mathrm{Beta}(\beta,\alpha)$, i.e. the above equations hold symmetrically for α and β interchanged, and n, l instead of m, k. This gives us the second required term to compute the specific heat at unit temperature,

$$\begin{aligned}
&\mathrm{E}[(1-r)^2\log^2(1-r)] \qquad (16)\\
&= \frac{\beta(\beta+1)\left((\psi_0(\beta+2)-\psi_0(\alpha+\beta+2))^2+\psi_1(\beta+2)-\psi_1(\alpha+\beta+2)\right)}{(\alpha+\beta)(\alpha+\beta+1)}.
\end{aligned}$$

Including derivatives w.r.t. both α and β, we more generally arrive at

$$\mathrm{E}[\log(r)^m r^k \log(1-r)^n (1-r)^l] = \frac{\prod_{i=0}^{k-1}(\alpha+i)\prod_{j=0}^{l-1}(\beta+j)}{\prod_{i=0}^{k+l-1}(\alpha+\beta+i)} g_{(m,n)}(\alpha+k,\beta+l).$$

We get recursive formulas for $g_{(m,n)}$, starting at $g_{(0,0)}(\alpha,\beta)=1$:

$$\begin{aligned}
g_{(m+1,n)}(\alpha,\beta) &= (\psi_0(\alpha)-\psi_0(\alpha+\beta))\, g_{(m,n)}(\alpha,\beta) + \frac{\delta}{\delta\alpha} g_{(m,n)}(\alpha+\beta) \\
g_{(m,n+1)}(\alpha,\beta) &= (\psi_0(\beta)-\psi_0(\alpha+\beta))\, g_{(m,n)}(\alpha,\beta) + \frac{\delta}{\delta\beta} g_{(m,n)}(\alpha+\beta).
\end{aligned}$$

To compute $c(T=1)$, we still require the case of $m=k=n=l=1$ given by

$$\mathrm{E}[r(1-r)\log(r)\log(1-r)] = \frac{\alpha\beta}{(\alpha+\beta)(\alpha+\beta+1)} g_{(1,1)}(\alpha+1,\beta+1), \tag{17}$$

with

$$\begin{aligned}
g_{(1,1)}(\alpha+k,\beta+l) = \psi_0(\alpha+k)\psi_0(\beta+l) - \psi_0(\alpha+\beta+k+l)\,(\psi_0(\alpha+k)+\psi_0(\beta+l)) \\
+ \psi_0(\alpha+\beta+k+l)^2 - \psi_1(\alpha+\beta+k+l).
\end{aligned} \tag{18}$$

Combining the results of equations (15), (16), (17), (18) with eq. (11), we arrive at

$$\begin{aligned}
\frac{c(T=1)}{n} &= \int_0^1 f(r)\,(\eta(r)-h)^2\,dr \\
&= \frac{\alpha(\alpha+1)\psi_1(\alpha+1)+\beta(\beta+1)\psi_1(\beta+1)}{(\alpha+\beta)(\alpha+\beta+1)} \\
&+ \frac{\alpha\beta\,(\psi_0(\alpha+1)-\psi_0(\beta+1))^2}{(\alpha+\beta)^2(\alpha+\beta+1)} - \psi_1(\alpha+\beta+1) \\
&= \mathrm{Var}[r]\,(\psi_0(\alpha+1)-\psi_0(\beta+1))^2 - \psi_1(\alpha+\beta+1).
\end{aligned}$$

3.3 Effects of firing rates

In our flat model analyses, we considered neural populations with various sizes and average correlation strengths and usually treated firing rates as fixed, generally at the rate of 1.5 Hz [16]. In general, however, different experimental stimuli may induce different average firing rates within the populations. Our analytic predictions from 3.1 and 3.2 for the divergence rate of specific heat can be evaluated for various combinations of average correlation and average firing rate (Fig. H). Our predictions show that for the systems with firing rates around 1.5 Hz, the specific heat divergence rates indeed depends on average firing rates for a wide range of correlations strengths, albeit weakly. Thus comparisons of exact specific heat values across different experimental conditions also need to take differences in firing rates into account.

3.4 Specific heat divergence at clamped firing rates

Changes in temperature T scale all model parameters of the maximum entropy model, and thus in general affect firing rates, correlations and population spike count statistics. For flat models, varying T during the specific heat analysis explores the behaviour through one-dimensional family of models parametrised by $\frac{1}{T}V \in \mathbb{R}^{n+1}$. Moving along

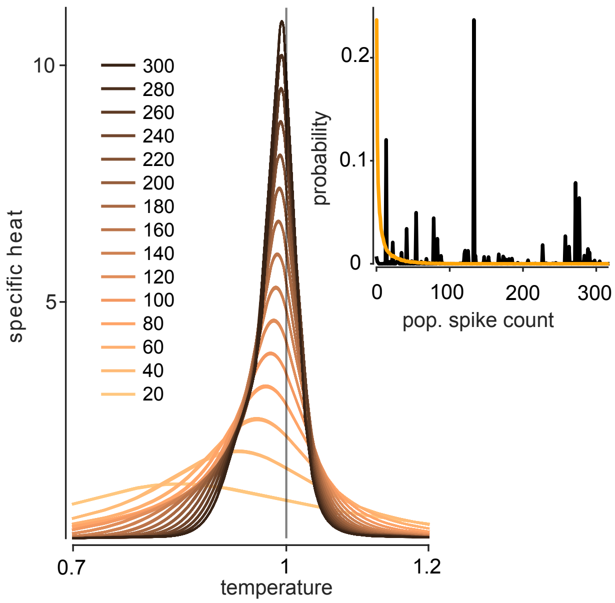

Figure G. Diverging specific heat for a non-natural spike-count distribution The values of the population spike count distribution $P(K)$ obtained from the retinal simulation with $N = 316$ in response to natural image stimulation (orange, inset) were shuffled (black trace, inset) across K, to yield a 'pathological' $P(K)$. We simulated data for this $P(K)$ from a flat model, and subsampled subpopulations of size $n = 20, \ldots, 300$. The specific heat traces computed from this data also diverges and has a peak at unit temperature

this trajectory, we can explore a range of possible average correlation strengths ρ between the entries of spike patterns $\mathbf{x} \in \mathbb{R}^n$: from the average correlation strength found in the data at $T = 1$, to usually larger ρ for $T \to 0$, and to $\rho \to 0$ for $T \to \infty$. However, not only the correlations ρ change with T, but the firing rate $\frac{1}{n}\mathrm{E}[K]$ is also affected.

We showed that the specific heat diverges whenever $T = 1$, and that it does not diverge otherwise. Is this also true if one 'clamps' firing rates such that they are not altered by changing T?

Tkačik et al. [16] introduced a modified temperature analysis for the K-pairwise maximum entropy model: After changing T, they numerically optimized the h parameters ($\tilde{h}_i(T)$) such that the firing rates were not affected by temperature:

$$\log P_T(\mathbf{x}|\lambda) = \sum_i \tilde{h}_i(T) x_i + \frac{1}{T}\left(\sum_{ij} J_{ij} x_i x_j + V_k\right) - \log Z(J, V, \tilde{h}(T)), \qquad (19)$$

for $k = \sum_{i=1}^n x_i$. The adjusted firing rate parameters $\tilde{h}_i(T)$ are recomputed for each temperature T and for each neuron i to keep the resulting firing rates $\mathrm{E}_{\frac{\lambda}{T}}[x_i]$ fixed to those firing rates obtained at $T = 1$.

We here show that for flat models, this result will be generically true: Specific heat diverges whenever $T = 1$, even for a modified analysis in which firing rates are clamped: The flat model assumes all $h_i = \bar{h}$, $J_{ij} = \bar{J}$ to be identical. Hence we can achieve such an altered temperature scaling for the flat model by introducing a linear term $\bar{h}(T)k$ to

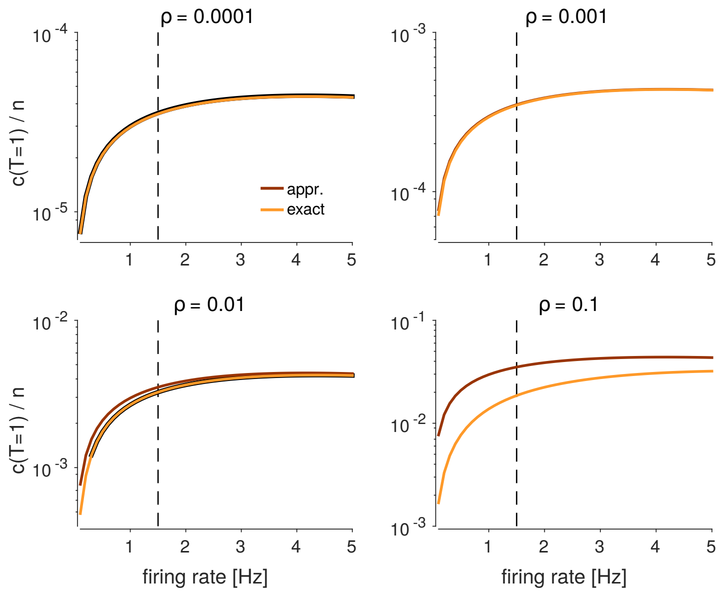

Figure H. Specific heat divergence and average firing rate in flat models Specific heat divergence rates $\tilde{c} = c(T = 1)/n$ in flat models for fixed average correlation strengths ρ and under various firing rates. Traces computed for general flat models using the approximate asymptotic prediction (12) (red, valid for small correlation strengths ρ), and for beta-binomial models using the asymptotically exact formula (19) (orange). Dashed vertical line marks 1.5 Hz, corresponding to the average firing rate of our retina simulation under stimulation with natural images.

the model log-likelihood,

$$\log P_T(k|V) = \frac{V_k}{T} - \bar{h}(T)k + \log \binom{n}{k} - \log Z(V, \bar{h}(T)). \tag{20}$$

The scalar factor $\bar{h}(T)$ for temperature T is chosen such that that the average firing rate $\frac{1}{n}\mathrm{E}_T[K]$ is identical to that of the data (at $T = 1$), i.e. $\mathrm{E}_T[K] = \mathrm{E}_1[K]$.

We repeated the specific heat analysis on our simulated RGC data with this adjustment. We found the same qualitative features in the specific heat traces obtained for different population sizes n: The peak of the curves diverges as the population size n is increased, and moves closer to unit temperature for increasing n (Fig. I). We also see that between the different experimental stimuli, there is an increase in the slope of the specific heat divergence from checkerboard stimuli (short-range spatial correlations, weak average correlations, Fig. Ia) to full-field flicker (infinite-range correlations, strong average correlations, Fig. Ic).

The derivations on specific heat divergence in section 2 also apply for the analysis with clamped firing rates: In particular, the specific heat will still diverge at unit temperature under the same conditions as before, since the original and the firing rate-preserving temperature scalings coincide at $T = 1$, i.e. $\bar{h}(T = 1) = 0$. For any other temperature $T = \frac{1}{\beta} \neq 1$, for fixed β, V, we can rewrite equation 20 as

$$P_\beta(k) = \frac{1}{Z_\beta}\binom{n}{k}\exp(V_k)^\beta \exp\left(-\bar{h}k\right) = \frac{1}{Z_\beta}\binom{n}{k}^{1-\beta}\exp(-\bar{h}k)^{1-\beta}P(k)^\beta,$$

where we dropped explicit dependencies of $P_T(k|V)$ on V and of $\bar{h}$ on β, as well as those

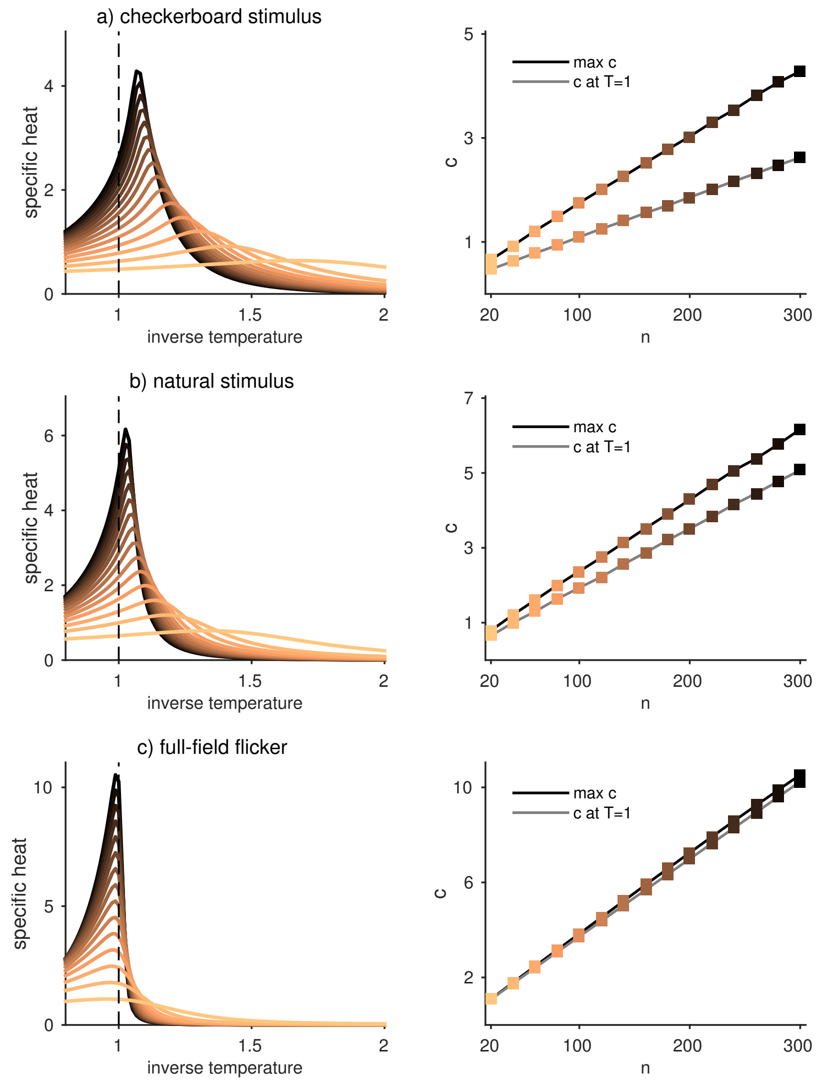

Figure I. Signatures of criticality in flat models with clamped firing rates Specific heat capacities computed from beta-binomial approximations to population spike-count distributions $P(K)$ with clamped firing rates. For each temperature T, we adjusted the model to keep the average firing rate fixed to the average firing rate of $T = 1$. Spike-count distributions for population sizes $n = 20, \ldots, 300$ for each n were obtained from 50 subpopulations drawn uniformly from our RGC simulation. Specific heat values are averages over subpopulations. Panels show averaged specific heat traces as a function of $\frac{1}{T}$ for each population n (left), and average specific heat values at peak and at $T = 1$ (right), for **a**) checkerboard stimuli, **b)** natural stimuli and **c)** full-field flicker stimuli.

of Z for notational clarity. In the large-n approximation $k = rn$, the above becomes

$$f_\beta(r) \approx \frac{n}{Z_\beta} \exp\left(n(1-\beta)\{\eta(r) - \bar{h}r\}\right) P(rn)^\beta.$$

The latter equation for large n again is dominated by the exponential term

$$\exp\left(n(1-\beta)\{\eta(r) - \bar{h}r\}\right) = \exp\left(n(1-\beta)\bar{\eta}(r)\right).$$

The inner function in r, $\bar{\eta}(r) = \eta(r) - \bar{h}r$, has its mode at $\bar{r} = \frac{1}{1+\exp(\bar{h})} \in]0,1[$. Plugging $\bar{r}$ into $\bar{\eta}(r)$ shows that the value attained at the mode is

$$\bar{\eta}(\bar{r}) = -\bar{h} + \log\left(1 + \exp\bar{h}\right),$$

which is zero if and only if $\bar{h} = \log\left(1 + \exp\bar{h}\right)$, i.e. if and only $\exp\bar{h} = 1 + \exp\bar{h}$ or $1 = 0$. Hence, $\bar{\eta}(\bar{r}) \neq 0$ for all $\bar{h} \in \mathbb{R}$ and all $\beta \neq 1$. Thus, for large n, either $f_\beta(r) \approx \delta(r - \bar{r})$, or $f_\beta(r) \approx \frac{1}{2}\delta(r-1) + \frac{1}{2}\delta(r-0)$, and the integral in the specific heat vanishes in either case.

4 Correlations under uniform subsampling

In previous sections, we derived the dependence of the specific heat divergence on correlation strength in populations.

For the methods developed in [16], uniformly random subsampling of n many neurons from a large fixed recording of size N was used to obtain several subpopulation at each population size. In terms of average correlations, we can formulate this subsampling process as selecting $n \times n$ principal submatrices from a large fixed $N \times N$ matrix describing the correlations of N many random variables. The average correlation of the subpopulation is the average over the respective selected entries. We denote the full index set for N many random variables as $[N] = \{1, \ldots, N\}$. Let $\mathcal{I}_n = \{I \subseteq [N] : |I| = n\}$ be the set of all size-n subpopulations of the full size-N recording. Let ρ_{ij} be the correlation between variables indexed with $i, j \in [N]$. We define the average correlation of a subpopulation $I \in \mathcal{I}_n$ as

$$\rho_n(I) := \frac{2}{n(n-1)} \sum_{i,j \in I, i<j} \rho_{ij} = \frac{1}{n(n-1)} \sum_{i,j \in I, i \neq j} \rho_{ij}.$$

We exclude diagonal entries $\rho_{ii} = 1 \ \forall i \in I$.

In the following sections, we derive some basic results on the behaviour of average correlation $\rho_n(I)$ for uniformly sampled subsets I of N many random variables. We are primarily interested in the mean $\mathrm{E}[\rho_n]$ and variance $\mathrm{Var}[\rho_n]$ of the average correlation with respect to the uniform distribution over subsets $P_n(I) = \binom{N}{n}^{-1}$. We will see that the mean $\mathrm{E}[\rho_n]$ is equal to the average correlation of the full recording $\rho_n([N])$ for all subpopulation sizes n– this is not surprising, as the entries of the sub-matrix are randomly sampled from $\rho_i j$, and characterize how the $\mathrm{Var}[\rho_n]$ decreases to zero with increasing n. Taken together, this predicts that in terms of average correlation, uniformly drawn subpopulations will quickly all behave like the full recording with increasing population size n, and the conditions for linear divergence of the specific heat derived in 2 are well-met even for sizes n that are actually experimentally accessible.

4.1 Scaling of average correlation with population size under uniform subsampling

Expected mean correlation: Under uniform subsampling, the expected mean correlation strength $\mathrm{E}[\rho_n]$ of populations of any size $n \leq N$ is equal to the average correlation $\rho_N([N])$ of the entire recording $[N]$, regardless of the exact correlation structure structure

$\{\rho_{ij}\}_{ij}, i \neq j$. This is simply a consequence of the fact that the entries of the submatrix are randomly (but not independently) drawn from the big correlation matrix. More formally:

Proof: The expected mean correlation across populations of fixed size n for uniformly drawn populations $\left(\forall I \in \mathcal{I}_n : P_n(I) = \binom{N}{n}^{-1}\right)$ is given by

$$\begin{aligned}
\mathrm{E}[\rho_n] &= \sum_{I \in \mathcal{I}_n} P_n(I)\rho_n(I) \\
&= \binom{N}{n}^{-1} \sum_{I \in \mathcal{I}_n} \rho_n(I) \\
&= \binom{N}{n}^{-1} \frac{2}{n(n-1)} \sum_{I \in \mathcal{I}_n} \sum_{i,j \in I, i<j} \rho_{ij} \\
&= \binom{N}{n}^{-1} \frac{2}{n(n-1)} \sum_{i,j \in [N], i<j} \binom{N-2}{n-2} \rho_{ij} \qquad (21) \\
&= \frac{2}{N(N-1)} \sum_{i,j \in [N], i<j} \rho_{ij} \\
&= \rho_N([N]). \qquad (22)
\end{aligned}$$

For line 21, we used that there are exactly $\binom{N-2}{n-2}$ many subsets $I \subseteq [N]$ of size n that contain a fixed pair of distinct variables $i, j \in [N], i \neq j$.

Variance of mean correlation: Characterising the variance is a bit more involved, as subsampling a principal matrix is not the same as drawing entries independently, so it is not a-priori clear that the variance will drop as $1/n$. However, under uniform subsampling, the variance of mean correlation $\mathrm{Var}[\rho_n]$ decreases with population size n at least as $\mathrm{Var}[\rho_n] \propto \frac{1}{n}$.

Proof: We begin with the second moment,

$$\begin{aligned}
\mathrm{E}[\rho_n^2] &= \sum_{I \in \mathcal{I}_n} P_n(I)\rho_n^2(I) \\
&= \binom{N}{n}^{-1} \sum_{I \in \mathcal{I}_n} \rho_n^2(I) \\
&= \binom{N}{n}^{-1} \sum_{I \in \mathcal{I}_n} \left(\frac{2}{n(n-1)} \sum_{i,j \in I, i<j} \rho_{ij} \right)^2 \\
&= \binom{N}{n}^{-1} \frac{4}{n^2(n-1)^2} \sum_{I \in \mathcal{I}_n} \sum_{i,j,k,l \in I : i<j, k<l} \rho_{ij}\rho_{kl}. \qquad (23)
\end{aligned}$$

The four-index inner sum in 23 is problematic because the number of unique indices $|\{i\} \cup \{j\} \cup \{k\} \cup \{l\}| =: q$ for any valid quartet i, j, k, l can range between two (if $i = k, j = l$) and four. We split the set of index quartets i, j, k, l depending on how many of the indices are unique. Let

$$S_q^2(I) = \sum_{i,j,k,l \in I, i<j, k<l, |\{i\} \cup \{j\} \cup \{k\} \cup \{l\}| = q} \rho_{ij}\rho_{kl}.$$

Conveniently,

$$\sum_{i,j,k,l\in I, i<j, k<l} \rho_{ij}\rho_{kl} = S_2^2(I) + S_3^2(I) + S_4^2(I)$$

and

$$\begin{aligned}\sum_{I\in\mathcal{I}_n} \sum_{i,j,k,l\in I: i<j, k<l} \rho_{ij}\rho_{kl} &= \sum_{I\in\mathcal{I}_n} S_4^2(I) + \sum_{I\in\mathcal{I}_n} S_3^2(I) + \sum_{I\in\mathcal{I}_n} S_2^2(I) \\ &= \binom{N-4}{n-4} S_4^2([N]) + \binom{N-3}{n-3} S_3^2([N]) + \binom{N-2}{n-2} S_2^2([N]). \end{aligned} \tag{24}$$

For the last equality, we again used that there are $\binom{N-q}{n-q}$ possible size-n subsets of $[N]$ that contain a specific set of q many unique indices.

Assuming $n \geq 4$, plugging 24 into 23 yields

$$\mathrm{E}[\rho_n^2] = \frac{4}{n(n-1)N(N-1)} \left(\frac{(n-2)(n-3)}{(N-2)(N-3)} S_4^2([N]) + \frac{(n-2)}{(N-2)} S_3^2([N]) + S_2^2([N]) \right).$$

Rewriting the squared first moment as

$$\mathrm{E}[\rho_n]^2 = \frac{4}{N^2(N-1)^2} \left(S_4^2([N]) + S_3^2([N]) + S_2^2([N]) \right) \tag{25}$$

allows to obtain

$$\begin{aligned}\mathrm{Var}[\rho_n] &= \mathrm{E}[\rho_n^2] - \mathrm{E}[\rho_n]^2 \\ &= m_4^2([N])\gamma_{4,N}(n) + m_3^2([N])\gamma_{3,N}(n) + m_2^2([N])\gamma_{2,N}(n), \end{aligned} \tag{26}$$

with

$$\begin{aligned} m_4^2([N]) &= \frac{4}{N(N-1)(N-2)(N-3)} S_4^2([N]), \\ m_3^2([N]) &= \frac{1}{N(N-1)(N-2)} S_3^2([N]), \\ m_2^2([N]) &= \frac{2}{N(N-1)} S_2^2([N]), \\ \gamma_{4,N}(n) &= \frac{(n-2)(n-3)}{n(n-1)} - \frac{(N-2)(N-3)}{N(N-1)}, \\ \gamma_{3,N}(n) &= \frac{4(n-2)}{n(n-1)} - \frac{4(N-3)}{N(N-1)}, \\ \gamma_{2,N}(n) &= \frac{2}{n(n-1)} - \frac{2}{N(N-1)}. \end{aligned}$$

The space of variance traces $\mathrm{Var}[\rho_n] \in \mathbb{R}^N$ lies within the span of the vectors $\gamma_{q,N}(n), q \in \{2,3,4\}$. It is $\gamma_{2,N} + \gamma_{3,N} + \gamma_{4,N} \equiv 0$ for all $N > 1$. The respective coordinates for a given $N \times N$ correlation matrix are given by the normalised summary statistics $m_q^2([N])$ of that particular correlation matrix (see Fig. J).

For a coarse bound on the scaling of variance of the mean correlation, we once more

rewrite

$$\mathrm{Var}[\rho_n] = d + \frac{e}{n-1} + \frac{f}{n(n-1)}, \tag{27}$$
$$d = \frac{4}{N-3}\left(\mathrm{E}[\rho_{ij}]^2 - m_3^2([N])\right) - \frac{2\mathrm{Var}[\rho_{ij}]}{(N-2)(N-3)},$$
$$e = \frac{4(N+1)}{N-3}\left(m_3^2([N]) - \mathrm{E}[\rho_{ij}]^2\right) + \frac{8\mathrm{Var}[\rho_{ij}]}{(N-2)(N-3)},$$
$$f = \frac{8N}{N-3}\left(\mathrm{E}[\rho_{ij}]^2 - m_3^2([N])\right) + \frac{2N(N-5)\mathrm{Var}[\rho_{ij}]}{(N-2)(N-3)},$$

where we expressed $m_4^2([N])$ through $\mathrm{E}[\rho_{ij}], \mathrm{Var}[\rho_{ij}]$ and $m_3^2([N])$ via eq. 25. The exact scaling behaviour of $\mathrm{Var}[\rho_n]$ for $n << N$ thus depends on the values for d, e, f, which for any given correlation structure $\{\rho_{ij}\}$ are fixed constants independent of n. The variance in any case decreases with n at least as fast as $\frac{1}{n-1}$. For

$$m_3^2([N]) \approx \mathrm{E}[\rho_{ij}]^2 - \frac{2\mathrm{Var}[\rho_{ij}]}{(N+1)(N-2)}, \tag{28}$$

we have $e \approx 0$ and obtain $\mathrm{Var}[\rho_n] \propto \frac{N(N-1)}{n(n-1)} - 1$, which initially decays as just as independent sampling of correlation matrix entries (i.e. $\propto \frac{1}{n(n-1)}$) before eventually decreasing to exactly zero for $n = N$. Decay accelerates as n approaches N because the uniform subsampling scheme draws variables without replacement. See figure J for an example with $N = 100$ and an exponentially decaying spatial correlation profile that leads to $d = -7.25\text{e-}7$, $e = -5.84\text{e-}5$, $f = 0.013$, i.e. $e << f$.

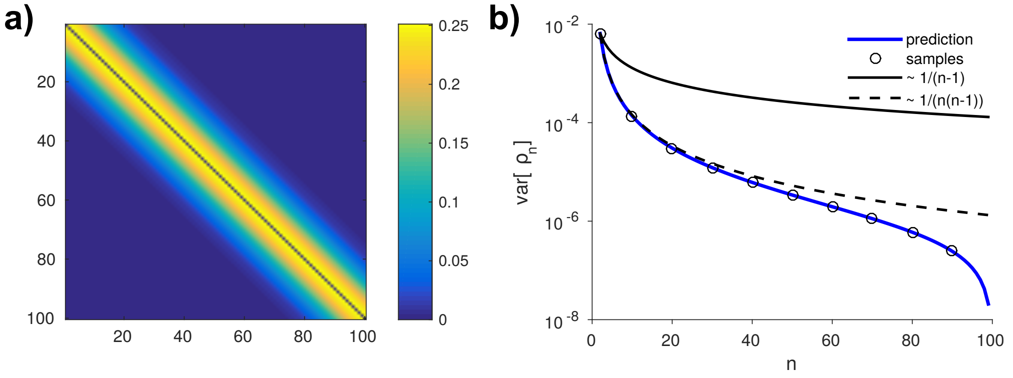

Figure J. Variance of mean for uniformly subsampled population of size n a) Example correlation matrix for $N = 100$ neurons, $\rho_{ij} = \frac{1}{0.2527}\exp\left(-\frac{|i-j|^2}{0.125}\right)$ (diagonal removed for displaying purposes). **b)** Variance of average correlation $\mathrm{Var}[\rho_n]$. Predictions from 4.1 with statistics $\mathrm{E}[\rho_{ij}], \mathrm{Var}[\rho_{ij}], m_3^2([N])$ computed from the matrix in **a**. Black circles give variance over empirical average correlations for 10000 populations uniformly drawn for each $n = 2, 10, 20, 30, \ldots, 90$.

4.2 Specific heat and non-random subsampling for the full K-pairwise model

We asked whether our analytical results on effects of the subsampling scheme for flat models also hold empirically for the more powerful K-pairwise models. To this end, we

trained K-pairwise models to in total 36 different populations obtained from ordered subsampling of our simulated RGCs (2 for each population size $n = 20, 40, \ldots 120$ for each of three stimulus conditions). As the K-pairwise model strongly restricts the maximal population size that we can feasibly work with, we did not use our full simulation for this analysis. Instead, we once randomly subsampled $N' = 120$ neurons out of the $N = 316$ simulated RGCs and subsequently treated this smaller subset as a proxy to the full recording. We point out that the largest population at $n = N'$ thus approximately shares the average firing rates and correlations of the full $N = 316$ recording (see section 4.1).

As with the flat models before, we then ordered the 120 remaining neurons according to their spatial location within the simulated patch of retina and subsequently added neighboring neurons to grow the populations, taking $n = 20, 40, \ldots 120$ for fitting K-pairwise models on the simulated RGC activity under checkerboard, natural and full-field flicker stimulation. We obtained two populations per populations size this way by once subsequently adding neurons traversing the retinal patch 'left-to-right' and once 'right-to-left'.

We summarise our results in Fig. K. As with the flat models before (Fig. 5f), the specific heat capacity at $T = 1$ no longer appears to diverge linearly for all three stimulus conditions. In particular, specific heat capacity growth slows down with larger n for the two conditions (checkerboard and natural images) that exhibit finite-length correlations within the stimuli. This again is consistent with decreasing average correlations within larger populations obtained by ordered subsampling (compare Fig. 5e).

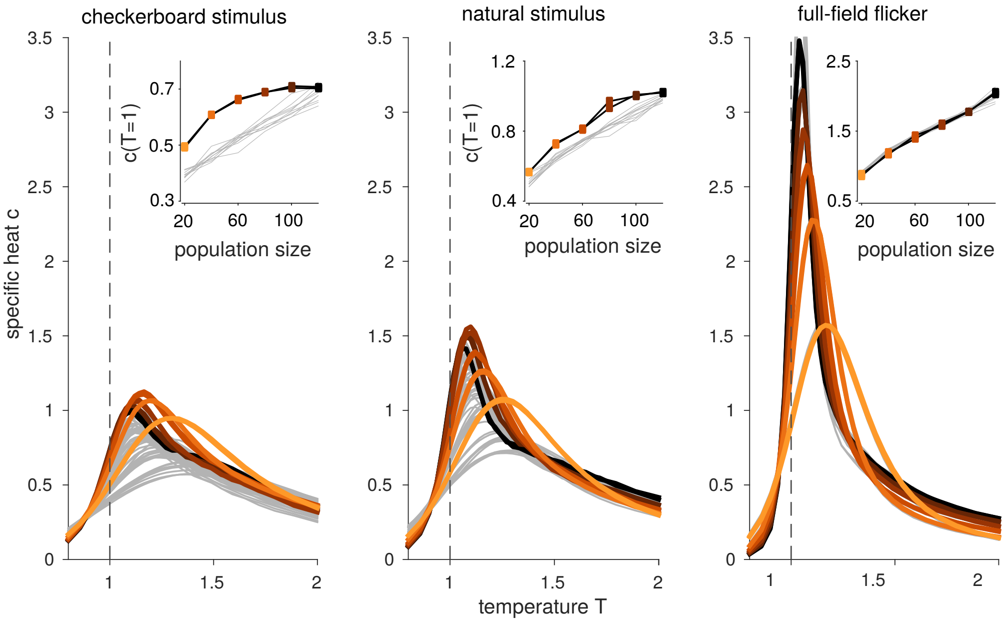

Figure K. Specific heat for K-pairwise models and ordered subsampling Specific heat capacity computed from K-pairwise models fit to subpopulations obtained from subsequently adding neighboring neurons (compare Fig. 5f). Colored lines give specific heat traces for populations of sizes $n = 20, 40, \ldots 120$. For each population size, two subpopulations were generated with ordered subsampling (corresponding to traversal of the simulated retinal patch once left-to-right and right-to-left). Grey lines give individual traces for 10 uniformly sampled populations for each of the six different population sizes (same as Fig. 4d), Insets: Specific heat at unit temperature.

References

1. Broderick T, Dudik M, Tkacik G, Schapire RE, Bialek W. Faster solutions of the inverse pairwise Ising problem. arXiv. 2007;0712.2437v2.

2. Rasmussen C, Williams C. Gaussian Processes for Machine Learning. MIT Press; 2006.

3. Broderick T. Construction of a pairwise Ising distribution over a large state space with sparse data. Princeton University; 2007.

4. Dudik M, Phillips SJ, Schapire RE. Performance guarantees for regularized maximum entropy density estimation. In: Learning Theory. Springer; 2004. p. 472–486.

5. Radhakrishna Rao C. Information and accuracy attainable in the estimation of statistical parameters. Bulletin of the Calcutta Mathematical Society. 1945;37(3):81–91.

6. Blackwell D. Conditional expectation and unbiased sequential estimation. The Annals of Mathematical Statistics. 1947;p. 105–110.

7. Berkson J. Tests of significance considered as evidence. Journal of the American Statistical Association. 1942;37(219):325–335.

8. Carlson D, Stinson P, Pakman A, Paninski L. Partition Functions from Rao-Blackwellized Tempered Sampling. arXiv preprint arXiv:160301912. 2016;.

9. Schmidt M. minFunc: unconstrained differentiable multivariate optimization in Matlab; 2005. `http://www.cs.ubc.ca/~schmidtm/Software/minFunc.html`.

10. Kirkpatrick S, Gelatt CD, Vecchi MP, et al. Optimization by simulated annealing. science. 1983;220(4598):671–680.

11. Schneidman E, Berry MJn, Segev R, Bialek W. Weak pairwise correlations imply strongly correlated network states in a neural population. Nature. 2006;440(7087):1007–12.

12. Amari Si, Nakahara H, Wu S, Sakai Y. Synchronous firing and higher-order interactions in neuron pool. Neural Computation. 2003;15(1):127–142.

13. Macke JH, Opper M, Bethge M. Common input explains higher-order correlations and entropy in a simple model of neural population activity. Physical Review Letters. 2011;106(20):208102.

14. Tkačik G, Marre O, Mora T, Amodei D, Berry II MJ, Bialek W. The simplest maximum entropy model for collective behavior in a neural network. Journal of Statistical Mechanics: Theory and Experiment. 2013;2013(03):P03011.

15. Archer E, Park IM, Pillow JW. Bayesian entropy estimation for countable discrete distributions. The Journal of Machine Learning Research. 2014;15(1):2833–2868.

16. Tkačik G, Mora T, Marre O, Amodei D, Palmer SE, Berry MJ, et al. Thermodynamics and signatures of criticality in a network of neurons. Proceedings of the National Academy of Sciences. 2015;112(37):11508–11513.
